# Lower urinary tract symptoms in people with prostate conditions: an exploratory, cross-sectional study

**DOI:** 10.1590/1980-220X-REEUSP-2026-0116en

**Published:** 2026-10-26

**Authors:** Pedro Henrique dos Santos Viana, Thiago Souza de Melo, Rafael Luis Bressani Lino, Aline Helena Appoloni Eduardo

**Affiliations:** 1 Universidade Federal de São Carlos, Programa de Pós-graduação em Enfermagem, São Carlos, SP, Brazil.; 2 Universidade Federal do Amazonas, Coari, AM, Brazil.; 3 Universidade Federal de São Paulo, Escola Paulista de Enfermagem, São Paulo, SP, Brazil.

**Keywords:** Lower Urinary Tract Symptoms, Nursing Care, Urology, Prostatic Diseases

## Abstract

**Objetivo:**

To analyze the specific characteristics and factors associated with lower urinary tract symptoms among individuals with prostate conditions. Method: An exploratory, cross-sectional study conducted between 2023 and 2025 at a university hospital in the interior of the state of São Paulo. Participants were men over 18 years of age with prostate conditions. Data were collected using a demographic questionnaire and the International Prostate Symptom Score and were analyzed using descriptive and inferential statistics, including exploratory analyses and negative binomial regression.

**Results:**

A total of 68 participants were included, with a mean age of 66.7 years. Symptoms of moderate intensity predominated (47.1%); among the symptoms assessed, weak urine stream, urinary frequency, and nocturia were the most prevalent. Advanced age, higher educational attainment, high body weight, and black or brown skin color were associated with greater symptom severity, while alcohol consumption and employment showed a protective effect.

**Conclusion:**

Storage and voiding symptoms predominated; the identification of factors that positively and negatively influence the severity of lower urinary tract symptoms and inform nursing assessment.

## INTRODUCTION

Lower urinary tract symptoms (LUTS) are common clinical manifestations in men, particularly among those with prostate conditions such as benign prostatic hyperplasia, chronic prostatitis, and suspected or confirmed prostate cancer. These symptoms have a direct impact on quality of life, self-perceived health, psychological morbidity, and healthcare costs, both for individuals and the government(, 2).

Furthermore, as a consequence of LUTS, there is an increased risk of falls among the elderly, an increased risk of urinary tract infections due to incomplete bladder emptying^(2)^, emotional distress—especially as a result of urinary incontinence—social isolation, and dissatisfaction in managing the symptoms^(3)^.

Clinically, LUTS have multifactorial origins, triggered by organic changes in the lower urinary tract, and are classified into storage symptoms (urgency, frequency, nocturia, and incontinence), voiding symptoms (hesitation, weak stream, intermittency, and straining), and post-void symptoms (terminal dribbling and sensation of incomplete emptying)^(1)^.

The main factors contributing to the development of LUTS in men include increased prostate volume, urethral obstruction, bladder abnormalities, and non-urological factors. Among these, non-modifiable characteristics—such as aging—and modifiable factors—such as obesity, metabolic syndrome, and lifestyle habits—are particularly noteworthy^(4)^.

Understanding the impact of these modifiable conditions and lifestyle factors is important because it can inform the development of nursing interventions to manage the non-urological causes of LUTS^(5)^. This can be achieved through educational activities that raise awareness about the importance of changing habits that impair urinary elimination, for example. However, there is a gap in our understanding of the associated factors capable of exacerbating LUTS among people with prostate conditions.

Various population-based studies conducted in Europe, Korea, and Malaysia demonstrated a high prevalence of LUTS in different countries, with the most common symptoms being nocturia, weak urine stream, and post-void dribbling(6–8). In Brazil, the Brazil LUTS study found, among 2,751 men, that the most frequent symptoms were nocturia, urinary frequency, and terminal dribbling; it also highlighted that the majority of men experience symptoms of moderate intensity^(9)^.

Although they are not exclusive conditions for the development of LUTS, prostatic changes—especially benign prostatic hyperplasia—are described in the literature as important factors associated with the onset of these symptoms^(10)^. However, the relationship between LUTS and prostate cancer is controversial, as some studies suggest an association between these variables, while others have not identified a significant correlation^(11,12)^.

Nursing plays a central role in the early assessment and management of LUTS, with interventions focused on pelvic floor exercises, catheterization, and lifestyle counseling^(1)^. In Ireland, the establishment of clinics led by specialist nurses has demonstrated effectiveness, safety, and cost savings by expanding access to urological care and preventing inappropriate treatments^(13,14)^. In Brazil, studies point to effective interventions but also gaps in knowledge and in the standardization of LUTS assessment by nurses^(15)^.

Nursing care based on the Nursing Process has proven to be cost-effective for people with STUI, as the nursing assessment identifies clinical indicators (risk factors, signs, and symptoms) that help nurses recognize impaired urinary elimination. Furthermore, a nursing assessment focused on identifying specific clinical indicators among individuals with prostate conditions enables the early identification of initial symptoms, which are generally more responsive to the treatments and care provided. It also facilitates timely referral to other healthcare professionals, preventing delays in care. Delays in identifying more complex or recurrent cases can result in worsening of the clinical condition, loss of renal function, and increased demand for diagnostic tests and high-cost interventions, generating significant impacts on the healthcare system^(2)^.

In this context, identifying the most common symptoms in the population can contribute to clinical management and aid in the risk stratification of patients with prostate conditions. Furthermore, analyzing the prevalence of symptoms and the factors associated with their severity can lead to greater diagnostic accuracy and improve the effectiveness of therapeutic interventions.

Accordingly, this study was based on the following research questions: What are the main lower urinary tract symptoms experienced by people with prostate conditions, and what factors are associated with an increase in the severity of lower urinary tract symptoms? Therefore, the objective of this study was to analyze the specific characteristics and factors associated with lower urinary tract symptoms among people with prostate conditions.

## METHOD

### Type of Study

This was an exploratory, cross-sectional study, reported in accordance with the Strengthening the Reporting of Observational Studies in Epidemiology (STROBE) guidelines.

### Study Location

The study was conducted at a university hospital affiliated with a public institution of higher education in a municipality in the interior of the state of São Paulo. At the hospital, the research was carried out in the outpatient care unit, which provides care in various specialties. Specifically, the study took place at the general urology outpatient clinic, which serves as the municipal referral center for urological care and clinical follow-up.

### Sample and Eligibility Criteria

Participants aged 18 years or older, of biological male sex, who presented with lower urinary tract symptoms resulting from prostatic changes were included, as determined by the researchers’ review of medical records to identify patient-reported LUTS or medical documentation. Participants who performed self-catheterization or used an indwelling urinary catheter were excluded. Participants were selected using convenience sampling from among patients who had been previously scheduled by the institution for outpatient care.

### Data Collection Procedures and Tools

Data collection took place between October 2023 and June 2025. Patients were recruited as they arrived for elective outpatient visits with their urologist. After their medical consultation, participants were invited to take part in the study, and upon their agreement, the research team collected data through interviews. The interviews took place in a private room located in the outpatient clinic and lasted 20 to 30 minutes. The questionnaires included sociodemographic and clinical information, as described below:

Sociodemographic and Clinical Characterization Form: developed by the authors to identify sociodemographic variables (age, race, marital status, education level, and employment status) and clinical variables (risk factors for the development of prostate diseases, comorbidities, surgeries, and hospitalizations during the past year).

International Prostate Symptom Score (IPSS): assesses the presence of urinary symptoms (urinary frequency, urinary urgency, nocturia, intermittent flow, weak urine stream, straining, and incomplete emptying), for a total of seven items. The frequency of each symptom is assessed using a six-point Likert scale, where zero means the symptom never occurs and five means the symptom occurs almost all the time. The IPSS includes an additional question regarding the respondent’s quality of life, ranging from one to six points, where a score of one is assigned to “excellent” and six to “very poor.” The final score was calculated by summing the seven items on urinary symptoms and ranges from zero to 35 points; the results were interpreted according to the following categories: mild symptoms (zero to seven points), moderate (eight to 19 points), and severe (20–35 points)^(16)^. This instrument was selected because it is considered an essential tool in clinical practice for assessing LUTS and is widely used in studies conducted around the world. It allows for standardized quantification of lower urinary tract symptoms, guides treatment, monitors disease progression, and assesses the impact on the patient’s quality of life^(12)^. The IPSS was validated for the Brazilian context in a sample of 281 individuals with prostate conditions, with high reliability as indicated by a Cronbach’s alpha of 0.816^(17)^.

### Ethical Considerations

In accordance with the resolutions of the National Health Council, this study was approved by the Research Ethics Committee under Opinion No. 7,308,106 and CAAE: 82826624.2.0000.5504.

### Study Variables and Data Analysis

The dependent variable of the study was the severity of prostate symptoms, measured by the IPSS, analyzed both categorically (mild, moderate, and severe symptoms) and numerically (0–35 points), with higher scores indicating greater symptom severity. The independent variables were sociodemographic and clinical factors obtained from the participant profile questionnaire.

The data were organized in an Excel® spreadsheet and analyzed using SPSS® and R®. The normality of the dependent variable was verified using the Shapiro-Wilk test (p > 0.05), and parametric analyses were performed. Quantitative variables (continuous and discrete) were described using the mean and standard deviation, while qualitative variables (categorical and nominal) were presented as absolute and relative frequencies. The frequency of lower urinary tract symptoms was assessed by analyzing absolute values and percentages, taking into account the prostatic conditions present in the sample and the intensity of urinary symptoms as identified by the IPSS.

To investigate the associations between the independent variables and the IPSS scores, we used Student’s t-test, ANOVA, and Pearson’s correlation. Considering the IPSS as a categorical variable, we used the Kruskal-Wallis test, Fisher’s exact test, and Pearson’s chi-square test. In addition, a negative binomial regression model was performed to estimate the effect of the independent variables on IPSS scores.

A ROC curve analysis was also conducted, using the area under the curve (AUC), to assess the accuracy of the IPSS in identifying mild and moderate/severe symptoms, with sensitivity and specificity calculated. A significance level of 5% and a 95% confidence interval were adopted.

## RESULTS

A total of 68 people participated in the study. During the recruitment process, six were excluded: one due to refusal to participate (n = 1) and five because they could not be contacted while waiting for their outpatient appointment (n = 5). The participants’ ages ranged from 41 to 92 years, with a mean of 66.7 years (standard deviation ±10.7). Table 1 presents the full set of characteristics of the study participants and the associations with the results obtained from the assessment of lower urinary tract symptom severity.

**Table 1:** Characterization of the sociodemographic and clinical variables of the study participants – São Carlos, SP, Brazil, 2023–2025.

Variables	Total IPSS n = 68	*p* value	IPSS			
			Mild n = 19	Moderate n = 32	Severe n = 17	*p* value
	n (%)		n (%)	n (%)	n (%)	
**Age (years) Mean (SD)** < 60 years ≥ 60 years	66.7 (±10.7)	0.14*	04 (20.0) 15 (31.2)	14 (70.0) 18 (37.5)	02 (10.0) 15 (31.2)	0.02**
**Race** White Black Mixed-race Other	42 (61.8) 10 (14.7) 15 (22.0) 01 (1.5)	**0.01** ^§^	14 (33.3) 03 (30.0) 01 (6.7) 01 (100)	19 (45.2) 05 (50.0) 08 (53.3) 00 (0.0)	09 (21.4) 02 (20.0) 06 (40.0) 00 (0.0)	0.31^§§^
**Marital status** Married Living with a partner Divorced Widowed Other	50 (73.5) 02 (2.9) 06 (8.8) 01 (1.5) 09 (13.2)	0.10^§^	– 17 (34.0) 00 (0.0) 01 (16.7) 00 (0.0) 01 (11.1)	– 22 (44.0) 01 (50.0) 05 (83.3) 00 (0.0) 06 (47.1)	– 11 (22.0) 01 (50.0) 00 (0.0) 01 (100) 02 (25.0)	0.20^§§^
**Education** Elementary school High school College or higher	41 (60.3) 23 (33.8) 04 (5.9)	0.70^§^	13 (31.7) 06 (26.1) 00 (0.0)	18 (43.9) 11 (47.8) 03 (75.0)	10 (24.4) 06 (26.1) 01 (25.0)	0.77^§§^
**Occupation** Employed Unemployed Other	17 (25.0) 04 (5.9) 47 (69.1)	0.93^§^	03 (17.6) 00 (0.0) 16 (34.0)	11 (64.7) 04 (100) 17 (36.2)	03 (17.6) 00 (0.0) 14 (29.8)	0.06^§§^
**Children** Yes No	65 (95.6) 03 (4.4)	0.88^++^	19 (29.2) 00 (0.0)	30 (46.2) 02 (66.7)	16 (24.6) 01 (33.3)	0.60^§§^
**Family history of prostate cancer** Yes No	14 (20.6) 54 (79.4)	0.69^++^	02 (14.3) 17 (31.5)	09 (64.3) 23 (42.6)	03 (21.4) 14 (25.9)	0.51^$^
**Smoking** Yes No	29 (42.6) 39 (57.4)	**0.01** ^++^	05 (17.2) 14 (35.9)	13 (44.8) 19 (48.7)	11 (37.9) 06 (15.4)	**0.05** ^$^
**Alcohol consumption** Yes No	27 (39.7) 41 (60.3)	0.92^++^	05 (18.5) 14 (34.1)	16 (59.3) 16 (39.0)	06 (22.2) 11 (26.8)	0.18^$^
**Hospitalization in the past year** Yes No	– 17 (25.0) 51 (75.0)	0.26^++^	– 04 (23.5) 15 (29.4)	– 07 (41.2) 25 (49.0)	– 06 (35.3) 11 (21.6)	0.62^§§^
**Previous surgeries** Yes No	46 (67.6) 22 (32.4)	0.39^++^	13 (28.3) 06 (27.3)	19 (41.3) 13 (59.1)	14 (30.4) 03 (13.6)	0.22^§§^
**Physical activity** Yes No	24 (35.3) 44 (64.7)	0.65^++^	07 (29.2) 12 (27.3)	12 (50.0) 20 (45.5)	05 (20.8) 12 (27.3)	0.80^$^
**Comorbidities** Yes No	48 (70.6) 20 (29.4)	0.58^++^	15 (31.2) 04 (20.0)	20 (41.7) 12 (60.0)	13 (27.1) 04 (20.0)	0.35^§§^
**High blood pressure** Yes No	33 (48.5) 35 (51.5)	0.43^++^	10 (30.3) 09 (25.7)	14 (42.4) 18 (51.4)	09 (27.3) 08 (22.9)	0.82^$^
**Heart disease** Yes No	09 (13.2) 59 (86.8)	0.91^++^	01 (11.1) 18 (30.5)	06 (66.7) 26 (44.1)	02 (22.2) 15 (25.4)	0.77^$^
**Diabetes** Yes No	25 (36.8) 43 (63.2)	0.72^++^	08 (32.0) 11 (25.6)	11 (44.0) 21 (48.8)	06 (24.0) 11 (25.6)	0.38^§§^
**Obesity** Yes No	54 (79.4) 14 (20.6)	0.42^++^	15 (27.8) 04 (28.6)	26 (48.1) 06 (42.9)	13 (24.1) 04 (28.6)	0.92^§§^
**Perceived health** Excellent Good Fair Poor Very poor	10 (14.7) 30 (44.1) 20 (29.4) 05 (7.4) 03 (4.4)	0.13^§^	06 (60.0) 07 (23.3) 04 (20.0) 01 (20.0) 01 (33.3)	02 (20.0) 16 (53.3) 10 (50.0) 02 (40.0) 02 (66.7)	02 (20.0) 07 (23.3) 06 (30.0) 02 (40.0) 00 (0.0)	0.44^$^

*Pearson’s correlation test,

^§^ANOVA,

^++^t-test,

^**^Kruskal-Wallis test,

^§§^Fisher’s exact test,

^$^Pearson’s chi-square test.

The mean IPSS score was 13.5 (standard deviation ±8.9); regarding symptom severity, 19 (27.9%) patients had mild symptoms, 32 (47.1%) had moderate symptoms, and 17 (25%) had severe symptoms. In the sample, only one (1.5%) participant scored zero, indicating an absence of urinary symptoms, and only one participant scored the maximum (35 points), which classifies the patient as having severe symptoms and the presence of all symptoms assessed by the IPSS. In contrast, 66 (97.0%) participants presented with at least one urinary symptom.

Upon analyzing the total score obtained on the IPSS, it was found that the most prevalent symptoms were urinary frequency (item 2), weak urine stream (item 5), and nocturia (item 7), with response percentages indicating the presence of these symptoms at 75.0% or higher. Meanwhile, the least frequent symptoms were incomplete bladder emptying (item 1), urinary hesitancy (item 3), and urinary straining (item 6), with response frequencies indicating the absence of symptoms at 51.5%, 54.5%, and 63.3%, respectively. In addition, the final question on the IPSS assesses participants’ quality of life, which ranged from good to moderate among all study participants. Figure 1 illustrates the frequencies of symptoms based on the total score and across different intensities (mild, moderate, and severe).

**Figure 1:**
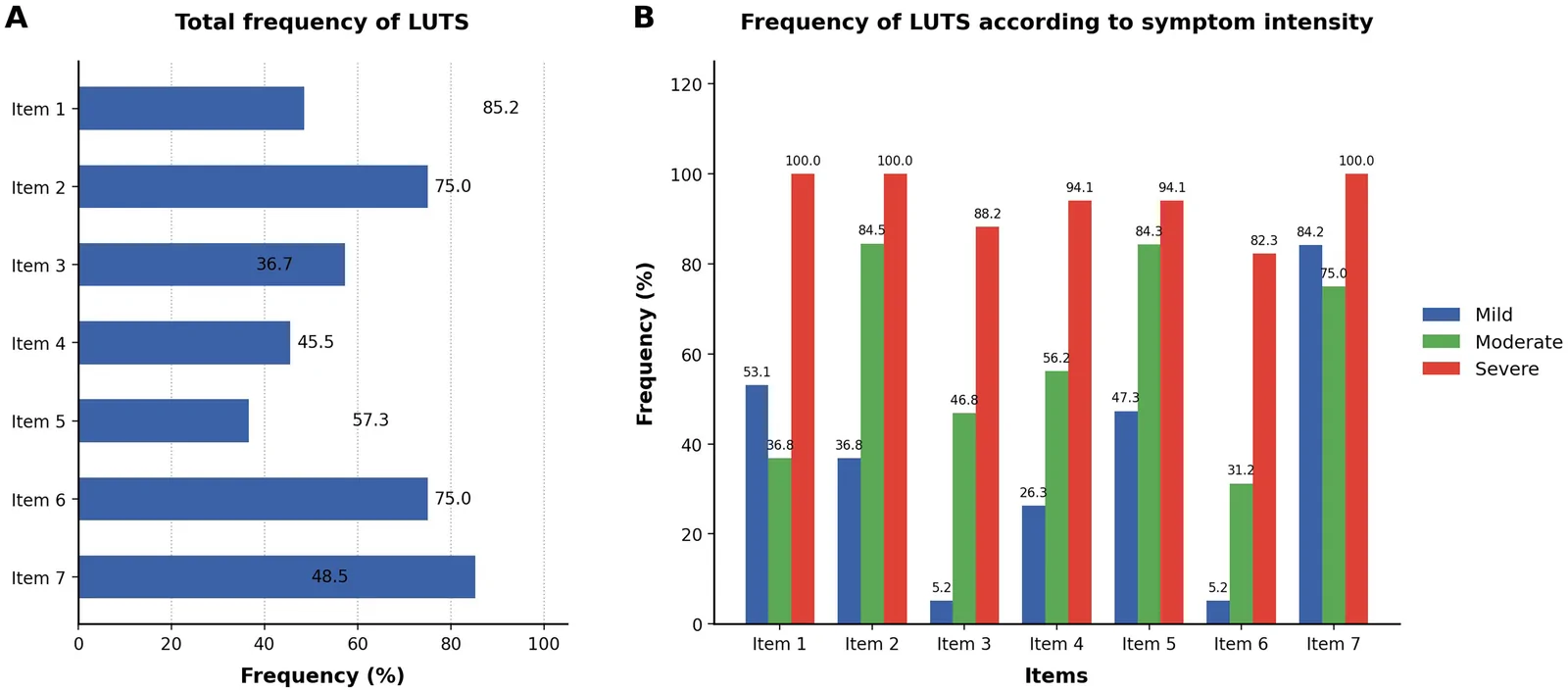
Frequency of lower urinary tract symptoms assessed by the IPSS.

Legend: Item 1. Incomplete bladder emptying; Item 2. Urinary frequency; Item 3. Urinary hesitancy; Item 4. Urinary urgency; Item 5. Weak urinary stream; Item 6. Urinary straining; Item 7. Nocturia.

The investigation of factors associated with symptom severity using a binomial analysis that employed a regression model revealed the influence of different variables on the worsening of symptom severity (Table 2).

**Table 2:** Negative binomial regression model with estimates of the effects of independent variables on the severity of STUI as assessed by the IPSS – São Carlos, SP, Brazil, 2023–2025.

Variables	Variable category	β (SE)	*p*-value	IRR	CI
Age	–	0.04 (0.01)	<0.001	1.04	1.02–1.06
Race	White Black Brown Others	– 0.41 (0.17) 0.63 (0.16) 0.08 (0.53)	– 0.02 <0.001 0.86	– 1.88 1.51 1.09	1.35–2.62 1.07–2.15 0.37–3.10
Family history of cancer	Yes No	-0.21 (0.11) –	0.07 –	0.81 –	0.63–1.03 –
Education level	Elementary School High School Higher Education	– 0.32 (0.12) 1.23 (0.30)	– 0.01 <0.001	– 1.39 3.43	– 1.05–1.84 1.95–6.11
Alcohol consumption	Yes No	-0.30 (0.13) –	0.02 –	0.77 –	0.56–0.96 –
High blood pressure	Yes No	0.25 (0.13) –	0.06 –	1.29 –	0.97–1.70 –
Weight	–	0.01 (0.00)	0.01	1.01	1.00–1.02
Children	Yes No	-0.49 (0.32) –	0.13 –	0.61 –	0.33–1.13 –
Occupation	Employed Unemployed Retired	– -0.40 (0.26) -0.54 (0.21)	– 0.12 0.001	– 0.67 0.58	– 0.39–1.14 0.38–0.87
Paid work	Yes No	0.32 (0.13) –	0.01 –	1.38 –	1.05–1.82 –

β = Estimated value, (SE) = Standard error, IRR = Incidence rate ratio, CI = Confidence interval. Stepwise criterion for variable selection. Total coefficient of determination: -1,12. Intercept (Standard error): -0.81 (0.80); p > 0.05.

The results of the regression analysis showed that increasing age was directly associated with more severe urinary symptoms, with each additional year representing an average 4% increase in the IPSS score (IRR = 1.04; 95% CI: 1.02–1.06; p < 0.001), participants who identified as mixed-race and Black had scores that were 51% (IRR = 1.51; 95% CI: 1.07–2.15; p < 0,001) and 88% (IRR = 1.88; 95% CI: 1.35–2.62; p = 0,02) compared to white participants; those with higher levels of education experienced more severe symptoms, both among those with a high school education (IRR = 1.39%; 95% CI: 1.05–1.84; p = 0.01) and those with a college education (IRR = 3.43; 95% CI: 1.95–6.11; p < 0.001), compared to those with elementary school education. In addition, body weight (p = 0.01) and engagement in paid work (p = 0.01) were also associated with increased symptom intensity.

Alcohol consumption was associated with a 27% reduction in the IPSS score (IRR = 0.77; p = 0.02), and being employed was associated with a 42.1% reduction in the IPSS score compared to retired participants (p = 0.001).

For identifying moderate and severe urinary symptoms, the IPSS showed a sensitivity of 91.7% and a specificity of 68.4%. The diagnostic accuracy for identifying differences in the severity of urinary symptoms was confirmed with an AUC of 0.865 (Figure 2).

**Figure 2:**
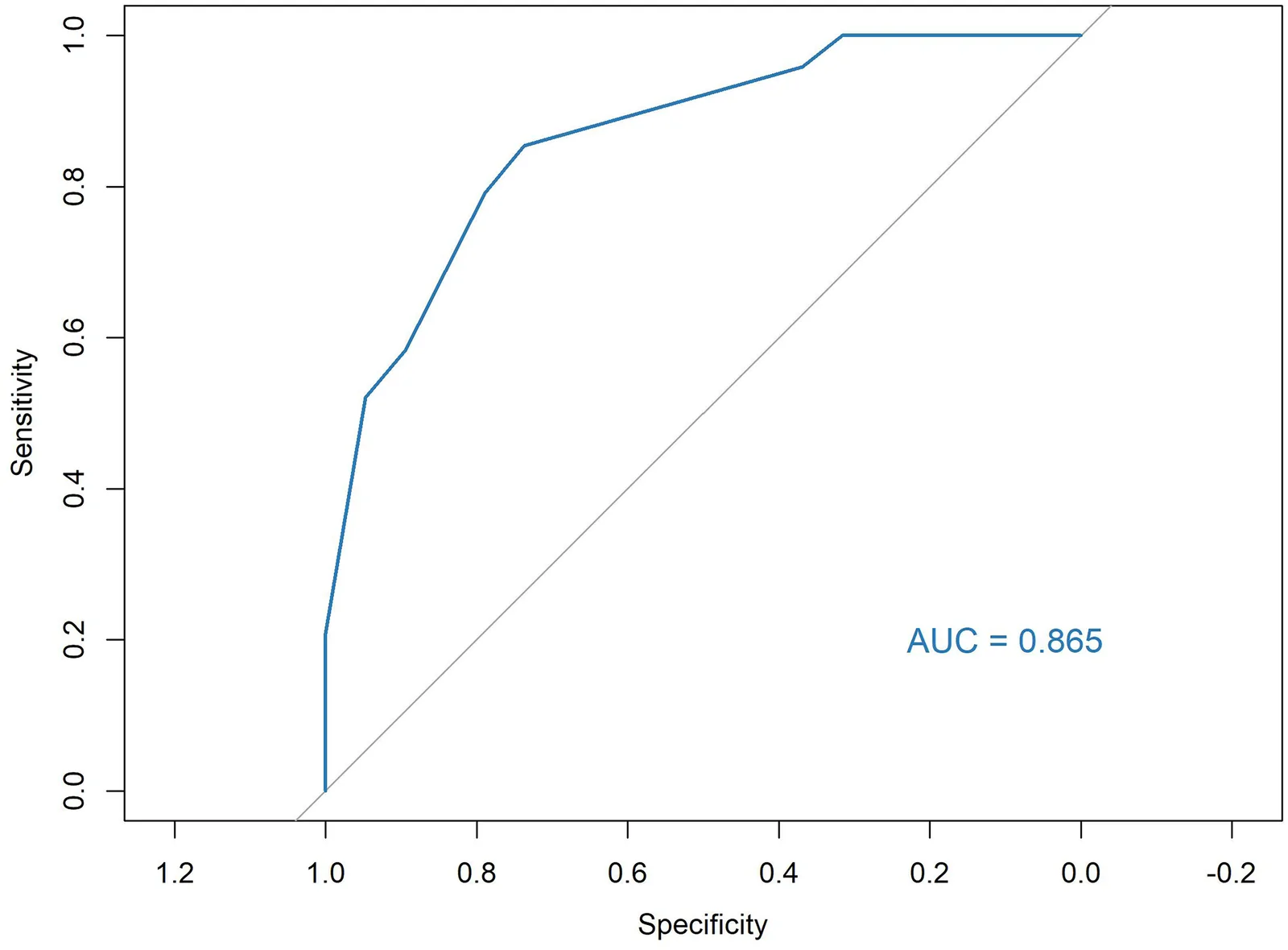
ROC curve for IPSS scores in relation to the discrimination between mild and moderate/severe levels.

## DISCUSSION

This study found that men with prostate conditions exhibited moderate levels of urinary symptoms and that urinary frequency, weak urine stream, and nocturia were the main symptoms reported. Factors associated with increased severity of LUTS in this population included advancing age, smoking, body weight, identifying as brown or Black, higher levels of education, and being engaged in paid work.

Moderate LUTS severity is common in the population with prostate conditions, as assessed by the IPSS^(12,18)^; this reinforces the prevalence of moderate symptoms in men aged 40 and older^(19)^. This finding is relevant for proposing interventions aimed at early management of LUTS among men, as these symptoms worsen over time and are associated with impairment in activities of daily living^(19)^, quality of life, and symptoms of anxiety and depression^(20)^.

Age is a determining factor in the worsening of urinary symptoms; in this study, a one-year increase in participants’ age corresponded to an approximately 4% increase in the IPSS score—that is, in symptom severity. These findings are consistent with those of studies conducted in different countries and involving patients with similar clinical conditions^(1,3,18)^. Given the population’s increasing longevity, the rising prevalence of symptoms with age, and the low rate of seeking care and adequate assessment of symptoms—especially among men with prostate conditions^(21)^—initiatives and investments that promote self-care among men are both important and necessary.

The way participants self-identified their race was a factor associated with higher levels of urinary symptoms; this finding is important for guiding the assessment of urinary symptoms in this population, given that research and comparisons regarding LUTS and prostate conditions among different ethnic groups are limited, and existing studies focus on racially homogeneous populations^(22)^.

Racial disparities regarding higher rates of prostate conditions and the severity of LUTS among Black individuals have been documented in the literature; therefore, studies must employ diverse population samples to reduce assessment biases among different racial groups^(22)^ and to develop effective measures to mitigate disparities in urologic care.

Within the field of scientific research on men’s health, the findings of this study align with research indicating a high prevalence of LUTS in older adult populations, but they also highlight that modifiable factors, such as smoking and weight gain, can exacerbate urinary symptoms and intensify urinary discomfort^(6,21,23)^. These results enable the implementation of interventions aimed at managing both conditions, with the goal of achieving outcomes such as a reduction in the discomfort caused by LUTS and an improvement in quality of life through actions that promote lifestyle changes.

Nevertheless, it is important to note that, unlike some studies demonstrating a consistent association between hypertension and diabetes mellitus and more severe symptoms^(23)^, in this study these variables did not show a statistically significant association, which reinforces the importance of considering the local context, the characteristics of the healthcare service, and the clinical profile of the study population.

The relationship between alcohol consumption and lower urinary tract symptoms remains inconsistent in the literature. Although the findings of this study suggest a possible mitigating effect of alcohol on LUTS, this association should be interpreted with caution, as it may reflect confounding bias related to the lack of distinction between moderate and excessive drinking patterns. In line with this finding, evidence suggests that moderate alcohol consumption may be associated with a reduction in urinary symptoms^(24)^. However, the same systematic review demonstrated that excessive alcohol consumption is associated with a significant worsening of LUTS, impairing urinary function—a finding that differs from the results observed in this study^(24)^.

In the present study, in addition to age and lifestyle habits, factors related to educational levels and employment status were also associated with increased LUTS severity. These findings support the hypothesis that there is a relationship between psychological stability—promoted by higher levels of education and income—and worsening LUTS scores^(25)^. These findings demonstrate the importance of a comprehensive assessment of individuals with LUTS, taking into account all social determinants of health, since the management of moderate-to-severe symptoms focuses on lifelong lifestyle modifications and the maintenance of a stable socioeconomic status, rather than on changes in short-time daily life^(25)^.

Furthermore, the association found in this sample between higher educational levels and worsening symptom intensity can be explained by the likelihood that participants had a better understanding of the instrument used to assess LUTS, which constitutes an information bias.

Both clinical guidelines and scientific evidence support conservative strategies in the care of individuals with prostate conditions^(1,26)^. In this approach, nursing plays a fundamental role, as it involves managing these symptoms, identifying risk factors, providing individualized counseling, and promoting behavioral changes^(2,14)^. The nurse’s proactive role, based on the Nursing Process, combined with continuous monitoring and the use of educational strategies, enhances patient adherence to the proposed measures, promoting not only symptom relief but also improved quality of life and the prevention of associated complications.

Thus, this study highlights the role of nurses as key players in the investigation, assessment, and follow-up of men with LUTS, as it clarifies the nature of urinary symptoms and identifies the demographic and behavioral factors that influence them. Recent literature emphasizes that nurses working in primary care can play an essential role in screening, counseling, and ensuring men’s adherence to urological follow-up^(26)^. It has been highlighted in the literature that training initiatives for urological nursing care have contributed to the consolidation of protocols for assessing urinary continence and voiding in the Brazilian context^(3)^.

Among the main scientific and practical contributions of this study is the provision of relevant evidence that strengthens the Nursing Assessment phase^(27)^, through a better understanding of the characteristics of urinary symptoms among people with prostate conditions, as well as their associated factors.

The limitations of this study center on the sample size, the cross-sectional design, and the non-probabilistic selection of participants in a specific hospital setting—factors that may introduce selection bias, limit the representativeness of the sample, and restrict the generalizability of the findings. Self-reported data collection is also subject to reporting bias, while certain statistical results should be interpreted with caution due to limitations inherent in the study’s statistical power. Furthermore, the lack of adjustment for potential relevant confounding variables may have influenced some of the observed associations. Thus, although the study provides important findings for urological care, the aforementioned limitations make it impossible to establish causal relationships between the variables investigated.

For future studies, we recommend the use of clinical variables detected through complementary tests for methodological triangulation and the follow-up of patients using longitudinal study designs to assess the effect of the investigated variables on the manifestation and intensity of urinary symptoms among individuals with prostate conditions.

Furthermore, expanding research that combines the use of validated tools, such as the IPSS, with supportive technologies, such as point-of-care ultrasound (POCUS), thereby strengthening nursing practice in healthcare settings^(11,28,29)^, can contribute to early diagnosis, risk stratification, and the provision of even more effective and personalized care for men with prostate conditions.

## CONCLUSION

This study achieved its objective by identifying the specific characteristics of lower urinary tract symptoms in individuals with prostate conditions treated at an outpatient clinic. A predominance of moderate-intensity symptoms was observed, particularly urinary frequency, weak urine stream, and nocturia.

Analysis of associated factors revealed that age, smoking, high body weight, higher educational attainment, paid employment, and black or brown skin color were related to greater symptom severity, while alcohol consumption and formal employment were associated with lower intensity of urinary symptoms.

Furthermore, in the study population, the IPSS demonstrated high sensitivity and specificity in distinguishing between mild, moderate, and severe symptoms—clinically relevant distinctions—and exhibited excellent accuracy.

These results reinforce the importance of considering demographic and behavioral factors during the nursing assessment of patients with prostate conditions, as they are related to the urinary symptoms presented.

## Data Availability

The entire dataset supporting the results of this study was published in the article itself.
