# Supplementary material for: Lower urinary tract symptoms in people with prostate conditions: an exploratory, cross-sectional study
Source: Rev Esc Enferm USP. 2026 Oct 26;60:e20260116. doi: 10.1590/1980-220X-REEUSP-2026-0116en (PMC13752870; doi:10.1590/1980-220X-REEUSP-2026-0116en)
Supplement: Translation

# Translation: Sintomas do trato urinário inferior em pessoas com condições prostáticas: estudo exploratório e transversal

**DOI:** 10.1590/1980-220X-REEUSP-2026-0116pt

**Authors:** Pedro Henrique dos Santos Viana, Thiago Souza de Melo, Rafael Luis Bressani Lino, Aline Helena Appoloni Eduardo

**Affiliations:** 1 Universidade Federal de São Carlos, Programa de Pós-graduação em Enfermagem, São Carlos, SP, Brasil.; 2 Universidade Federal do Amazonas, Coari, AM, Brasil.; 3 Universidade Federal de São Paulo, Escola Paulista de Enfermagem, São Paulo, SP, Brasil.

**Keywords:** Sintomas do Trato Urinário Inferior, Cuidados de Enfermagem, Urologia, Doenças Prostáticas

## Abstract

**Objetivo:**

Analisar as especificidades e os fatores associados aos sintomas do trato urinário inferior entre pessoas com condições prostáticas.

**Método:**

Estudo exploratório e transversal realizado entre 2023 e 2025 em um hospital universitário do interior do Estado de São Paulo. Participaram homens maiores de 18 anos, com alterações prostáticas. Os dados foram coletados por formulário de caracterização e pelo Escore Internacional de Sintomas Prostáticos, foram analisados pela estatística descritiva e inferencial, com análises exploratórias e regressão binomial negativa.

**Resultados:**

Incluíram-se 68 participantes, com média de idade de 66,7 anos. Houve predomínio dos sintomas de intensidade moderada (47,1%); considerando o conjunto dos sintomas avaliados, jato fraco, frequência urinária e noctúria foram os mais prevalentes. Idade avançada, maior escolaridade, peso corporal elevado e cor da pele preta ou parda associaram-se à maior gravidade, enquanto consumo de álcool e ocupação laboral mostraram efeito protetor.

**Conclusão:**

Houve predominância dos sintomas de armazenamento e esvaziamento, foram identificados fatores que influenciam positiva e negativamente a intensidade dos sintomas do trato urinário inferior, o que qualifica a avaliação de Enfermagem.

## INTRODUÇÃO

Os sintomas do trato urinário inferior (STUI) representam manifestações clínicas frequentes em homens, sobretudo entre aqueles com condições prostáticas, como hiperplasia prostática benigna, prostatite crônica e câncer de próstata suspeito ou confirmado. Esses sintomas repercutem diretamente na qualidade de vida, na autopercepção de saúde, na morbidade psicológica e nos custos em saúde, tanto individuais quanto governamentais^(1,2)^.

Ainda como consequência da ocorrência dos STUI, há aumento do risco de quedas entre os idosos, aumento do risco de infecção do trato urinário devido ao esvaziamento incompleto da bexiga^(2)^, constrangimento emocional, especialmente em decorrência da incontinência urinária, isolamento social e descontentamento em lidar com os sintomas^(3)^.

Clinicamente, os STUI são de origem multifatorial, desencadeados por alterações orgânicas do trato urinário inferior e são classificados em sintomas de armazenamento (urgência, frequência, noctúria e incontinência), de esvaziamento (hesitação, jato fraco, intermitência e esforço miccional) e pós-miccionais (gotejamento terminal e sensação de esvaziamento incompleto)^(1)^.

Os principais fatores para o desenvolvimento dos STUI em homens incluem o aumento do volume prostático, a obstrução uretral, alterações vesicais e fatores não urológicos. Entre estes, destacam-se as características não modificáveis, como o envelhecimento, e as modificáveis, como a obesidade, síndrome metabólica e hábitos de vida^(4)^.

O conhecimento do impacto destas condições modificáveis e do estilo de vida é importante, porque podem subsidiar o desenvolvimento de intervenções de Enfermagem para controlar a origem não urológica dos STUI^(5)^, por meio de atividades educativas que conscientizem as pessoas quanto à importância de mudanças de hábitos que comprometam a eliminação urinária, por exemplo. No entanto, evidencia-se uma lacuna acerca do que se conhece sobre os fatores associados capazes de intensificar os STUI entre as pessoas com condições prostáticas.

Diferentes estudos populacionais, realizados na Europa, Coreia e Malásia evidenciaram a alta prevalência dos STUI em diferentes países, os sintomas mais comuns foram noctúria, jato fraco e gotejamento pós-miccional^(6,7,8)^. No Brasil, o estudo Brasil LUTS verificou entre 2.751 homens que os sintomas mais frequentes foram noctúria, frequência urinária e gotejamento terminal, além de destacar que a maioria dos homens apresenta sintomas de intensidade moderada^(9)^.

Embora não sejam condições exclusivas para o desenvolvimento dos STUI, as alterações prostáticas, especialmente a hiperplasia prostática benigna, são descritas na literatura como importantes fatores associados ao surgimento desses sintomas^(10)^. Entretanto, a relação entre STUI e câncer de próstata é controversa, pois alguns estudos apontam a existência de associação entre essas variáveis, mas outros não identificaram correlação significativa^(11,12)^.

A Enfermagem desempenha papel central na avaliação precoce e no manejo dos STUI, com intervenções voltadas para os exercícios do assoalho pélvico, cateterismo e aconselhamento sobre estilo de vida^(1)^. Na Irlanda, a implantação de clínicas lideradas por enfermeiros especialistas demonstrou eficácia, segurança e redução de custos, ao ampliarem o acesso ao cuidado urológico e evitarem tratamentos inadequados^(13,14)^. No Brasil, estudos apontam intervenções eficazes, mas também lacunas no conhecimento e na padronização da avaliação dos STUI por enfermeiros^(15)^.

A assistência de Enfermagem fundamentada no Processo de Enfermagem mostra-se custo-efetiva para pessoas com STUI, pois por meio da avaliação de Enfermagem serão detectados indicadores clínicos (fatores de risco e sinais e sintomas) que auxiliam o enfermeiro a reconhecer o comprometimento da eliminação urinária. Além disso, a avaliação de Enfermagem direcionada à identificação de indicadores clínicos específicos entre as pessoas com condições prostáticas possibilita a identificação precoce dos sintomas iniciais, geralmente mais sensíveis aos tratamentos e cuidados implementados, além de favorecer o encaminhamento oportuno para outros profissionais de saúde, prevenindo atrasos no cuidado. A demora na identificação de casos mais complexos ou recorrentes pode resultar em agravamento do quadro clínico, perda da função renal e maior demanda por exames diagnósticos e intervenções de alto custo, gerando impactos significativos para o sistema de saúde^(2)^.

Nesse contexto, a identificação dos sintomas mais frequentes na população pode contribuir para o manejo clínico e auxiliar na estratificação de risco de pacientes com condições prostáticas. Ademais, a análise da prevalência dos sintomas e dos fatores associados à sua intensidade pode favorecer maior acurácia diagnóstica e aprimorar a efetividade das intervenções terapêuticas.

Deste modo, este estudo ancorou-se nas questões de pesquisa: Quais são os principais sintomas do trato urinário inferior manifestados por pessoas com condições prostáticas e quais são os fatores associados à piora da intensidade dos sintomas do trato urinário inferior? Portanto, o objetivo deste estudo foi analisar as especificidades e os fatores associados aos sintomas do trato urinário inferior entre pessoas com condições prostáticas.

## MÉTODO

### Tipo de Estudo

Tratou-se de um estudo exploratório e transversal, reportado conforme as diretrizes do Strengthening the Reporting of Observational Studies in Epidemiology (STROBE).

### Local do Estudo

O estudo foi realizado junto ao Hospital Universitário vinculado a uma instituição pública de ensino superior em um município no interior do Estado de São Paulo. No hospital, a pesquisa foi realizada no setor ambulatorial, que atende diversas especialidades. De maneira específica, o estudo ocorreu junto ao ambulatório de urologia geral, que é referência municipal no atendimento e no acompanhamento clínico em urologia.

### Amostra e Critérios de Elegibilidade

Foram incluídos participantes maiores de 18 anos, do sexo biológico masculino, que apresentavam sintomas do trato urinário inferior em decorrência de alterações prostáticas, conforme análise pelos pesquisadores do prontuário para identificar o relato de STUI pelo paciente ou registro médico. Foram excluídos os participantes que realizavam autocateterismo vesical ou faziam uso de sonda vesical de demora. A seleção dos participantes ocorreu por amostragem de conveniência, a partir dos pacientes previamente agendados pela instituição para atendimento ambulatorial.

### Procedimentos e Instrumentos para Coleta de Dados

A coleta de dados ocorreu entre outubro de 2023 e junho de 2025. Os pacientes foram recrutados conforme compareciam para atendimento ambulatorial, de forma eletiva, com o médico urologista. Após consulta médica, os participantes eram convidados a participar do estudo e diante da manifestação de aceite, a equipe de pesquisa realizava a coleta de dados por meio de entrevistas. As entrevistas aconteciam em uma sala reservada, localizada no ambulatório, com duração de 20 a 30 minutos. Os instrumentos contemplavam informações sociodemográficas e clínicas, conforme descritos a seguir:

Formulário de caracterização sociodemográfica e clínica: elaborado pelos autores, com o intuito de identificar as variáveis sociodemográficas (idade, cor, estado civil, escolaridade e situação de trabalho) e variáveis clínicas (fatores de risco para desenvolvimento de doenças prostáticas, comorbidades, cirurgias e internações realizadas no último ano).

Escore Internacional de Sintomas Prostáticos (International Prostate Symptom Score – IPSS): avalia a presença de sintomas urinários (frequência urinária, urgência urinária, noctúria, intermitência, jato urinário fraco, esforço e esvaziamento incompleto), que totaliza sete itens. A frequência de ocorrência de cada sintoma é avaliada por uma escala Likert de seis pontos, na qual zero significa que o sintoma não ocorre nunca e cinco que o sintoma ocorre quase todas as vezes. O IPSS possui uma questão adicional sobre a qualidade de vida do respondente, variando de um a seis pontos, na qual a pontuação um é atribuída à resposta excelente e seis péssima. O escore final se deu pela somatória dos sete itens sobre os sintomas urinários, e varia de zero a 35 pontos, a interpretação dos resultados se deu pela seguinte estratificação: sintomas leves (zero a sete pontos), moderados (oito a 19 pontos) e graves (20 a 35 pontos)^(16)^. Este instrumento foi selecionado por ser considerado uma ferramenta essencial na prática clínica para avaliação dos STUI e amplamente empregado em estudos desenvolvidos ao redor do mundo. O mesmo permite uma quantificação padronizada dos sintomas do trato urinário inferior, orienta o tratamento, monitora a progressão da doença e avalia o impacto dos sintomas na qualidade de vida do paciente^(12)^. O IPSS foi validado para o contexto brasileiro em uma amostra de 281 pessoas com condições prostáticas, com alta confiabilidade obtida pelo Alfa de Cronbach igual a 0,816^(17)^.

### Aspectos Éticos

Para atender às resoluções do Conselho Nacional de Saúde, o trabalho foi aprovado pelo Comitê de Ética em Pesquisa sob o parecer no 7.308.106 e CAAE 82826624.2.0000.5504.

### Variáveis do Estudo e Análise dos Dados

A variável dependente do estudo foi a intensidade dos sintomas do trato urinário inferior, mensurada pelo IPSS, analisado tanto de forma categórica (sintomas leves, moderados e graves) quanto numérica (0 a 35 pontos), sendo escores mais elevados indicativos de maior intensidade dos sintomas. As variáveis independentes corresponderam aos fatores sociodemográficos e clínicos obtidos por meio do formulário de caracterização dos participantes.

Os dados foram organizados em planilha eletrônica do Excel® e analisados nos programas SPSS® e R®. A normalidade da variável dependente foi verificada pelo teste de Shapiro-Wilk (p > 0,05), adotando-se análises paramétricas. Variáveis quantitativas (contínuas e discretas) foram descritas por média e desvio-padrão, enquanto variáveis qualitativas (categóricas e nominais) foram apresentadas em frequências absolutas e relativas. A avaliação da frequência dos sintomas do trato urinário inferior ocorreu pela análise dos valores absolutos e porcentagem, considerando as condições prostáticas apresentadas na amostra e a intensidade dos sintomas urinários identificada pelo IPSS.

Para investigação das associações entre as variáveis independentes e os escores do IPSS, empregaram-se teste t de Student, ANOVA e correlação de Pearson. Considerando o IPSS como variável categórica, utilizaram-se os testes de Kruskal-Wallis, Exato de Fisher e Qui-quadrado de Pearson. Adicionalmente, foi realizado modelo de regressão binomial negativa para estimar o efeito das variáveis independentes sobre os escores do IPSS.

Também foi conduzida análise da curva ROC, por meio da area under the curve (AUC), para avaliar a acurácia do IPSS na identificação de sintomas leves e de sintomas moderados/graves, sendo calculadas sensibilidade e especificidade. Adotou-se um nível de significância de 5% e intervalo de confiança de 95%.

## RESULTADOS

Participaram do estudo 68 pessoas. Durante o processo de recrutamento, seis não foram incluídas, sendo uma por recusa em participar (n = 1) e cinco por impossibilidade de contato no momento da espera para a consulta ambulatorial (n = 5). A idade dos participantes variou entre 41 e 92 anos, com média de 66,7 anos (Desvio-padrão ± 10,7). Na Tabela 1 está apresentada a totalidade das características dos participantes do estudo e as associações com os resultados obtidos com a avaliação da intensidade dos sintomas do trato urinário inferior.

**Tabela 1:** Caracterização das variáveis sociodemográficas e clínicas dos participantes do estudo – São Carlos, SP, Brasil, 2023–2025.

Variáveis	IPSS total n = 68	*p* valor	IPSS			
			Leve n = 19	Moderado n = 32	Grave n = 17	*p* valor
	n (%)		n (%)	n (%)	n (%)	
**Idade (anos) Média (DP)** < 60 anos ≤ 60 anos	66,7 (±10,7)	0,14^*^	04 (20,0) 15 (31,2)	14 (70,0) 18 (37,6)	02 (10,0) 15 (31,2)	0,02^**^
**Cor** Brancos Pretos Pardos Outro	42 (61,8) 10 (14,7) 15 (22,0) 01 (1,5)	**0,01** ^§^	14 (33,3) 03 (30,0) 01 (6,7) 01 (100)	19 (45,2) 05 (50,0) 08 (53,3) 00 (0,0)	09 (21,4) 02 (20,0) 06 (40,0) 00 (0,0)	0,31^§§^
**Estado civil** Casado Amasiado Divorciado Viúvo Outro	50 (73,5) 02 (2,9) 06 (8,8) 01 (1,5) 09 (13,2)	0,10^§^	– 17 (34,0) 00 (0,0) 01 (16,7) 00 (0,0) 01 (11,1)	– 22 (44,0) 01 (50,0) 05 (83,3) 00 (0,0) 06 (47,1)	– 11 (22,0) 01 (50,0) 00 (0,0) 01 (100) 02 (25,0)	0,20^§§^
**Escolaridade** Ensino fundamental Ensino médio Ensino superior	41 (60,3) 23 (33,8) 04 (5,9)	0,70^§^	13 (31,7) 06 (26,1) 00 (0,0)	18 (43,9) 11 (47,8) 03 (75,0)	10 (24,4) 06 (26,1) 01 (25,0)	0,77^§§^
**Ocupação** Empregado Desempregado Aposentados	17 (25,0) 04 (5,9) 47 (69,1)	0,93^§^	03 (17,6) 00 (0,0) 16 (34,0)	11 (64,7) 04 (100) 17 (36,2)	03 (17,6) 00 (0,0) 14 (29,8)	0,06^§§^
**Filhos** Sim Não	65 (95,6) 03 (4,4)	0,88^++^	19 (29,2) 00 (0,0)	30 (46,2) 02 (66,7)	16 (24,6) 01 (33,3)	0,60^§§^
**História de câncer de próstata na família** Sim Não	14 (20,6) 54 (79,4)	0,69^++^	02 (14,3) 17 (31,5)	09 (64,3) 23 (42,6)	03 (21,4) 14 (25,9)	0,51^$^
**Tabagismo** Sim Não	29 (42,6) 39 (57,4)	**0,01++**	05 (17,2) 14 (35,9)	13 (44,8) 19 (48,7)	11 (37,9) 06 (15,4)	**0,05** ^$^
**Consumo de bebida alcoólica** Sim Não	27 (39,7) 41 (60,3)	0,92^++^	05 (18,5) 14 (34,1)	16 (59,3) 16 (39,0)	06 (22,2) 11 (26,8)	0,18^$^
**Internação último ano** Sim Não	– 17 (25,0) 51 (75,0)	0,26^++^	– 04 (23,5) 15 (29,4)	– 07 (41,2) 25 (49,0)	– 06 (35,3) 11 (21,6)	0,62^§§^
**Cirurgias anteriores** Sim Não	46 (67,6) 22 (32,4)	0,39^++^	13 (28,3) 06 (27,3)	19 (41,3) 13 (59,1)	14 (30,4) 03 (13,6)	0,22^§§^
**Atividade física** Sim Não	24 (35,3) 44 (64,7)	0,65^++^	07 (29,2) 12 (27,3)	12 (50,0) 20 (45,5)	05 (20,8) 12 (27,3)	0,80^$^
**Comorbidade** Sim Não	48 (70,6) 20 (29,4)	0,58^++^	15 (31,2) 04 (20,0)	20 (41,7) 12 (60,0)	13 (27,1) 04 (20,0)	0,35^§§^
**Hipertensão arterial** Sim Não	33 (48,5) 35 (51,5)	0,43^++^	10 (30,3) 09 (25,7)	14 (42,4) 18 (51,4)	09 (27,3) 08 (22,9)	0,82^$^
**Cardiopatias** Sim Não	09 (13,2) 59 (86,8)	0,91^++^	01 (11,1) 18 (30,5)	06 (66,7) 26 (44,1)	02 (22,2) 15 (25,4)	0,77^$^
**Diabetes** Sim Não	25 (36,8) 43 (63,2)	0,72^++^	08 (32,0) 11 (25,6)	11 (44,0) 21 (48,8)	06 (24,0) 11 (25,6)	0,38^§§^
**Obesidade** Sim Não	54 (79,4) 14 (20,6)	0,42^++^	15 (27,8) 04 (28,6)	26 (48,1) 06 (42,9)	13 (24,1) 04 (28,6)	0,92^§§^
**Percepção da saúde** Excelente Boa Moderada Ruim Muito ruim	10 (14,7) 30 (44,1) 20 (29,4) 05 (7,4) 03 (4,4)	0,13^§^	06 (60,0) 07 (23,3) 04 (20,0) 01 (20,0) 01 (33,3)	02 (20,0) 16 (53,3) 10 (50,0) 02 (40,0) 02 (66,7)	02 (20,0) 07 (23,3) 06 (30,0) 02 (40,0) 00 (0,0)	0,44^§§^

^*^Teste de correlação de Pearson

^§^ANOVA

^++^Teste-t

^**^Teste de Kruskal-Wallis

^§§^Teste exato de Fisher

^$^Teste Qui-quadrado de Pearson.

A média do IPSS foi 13,5 (Desvio-padrão ± 8,9) e em relação à intensidade dos sintomas, 19 (27,9%) pacientes apresentaram sintomas leves, 32 (47,1%) sintomas moderados e 17 (25,0%) sintomas graves. Na amostra, somente um (1,5%) participante obteve o escore zero, que indica ausência de sintomas urinários e também apenas um participante obteve o escore máximo (35 pontos), que classifica com sintomas de intensidade grave e com a presença de todos os sintomas avaliados pelo IPSS. Em contrapartida, 66 (97,0%) participantes apresentaram ao menos um sintoma urinário.

Ao se analisar o resultado total da pontuação obtida pelo IPSS, verificou-se que os sintomas mais prevalentes foram frequência urinária (item 2), jato fraco (item 5) e noctúria (item 7), com percentuais de respostas que indicaram a presença destes sintomas com valor igual ou superior a 75,0%, enquanto os sintomas menos frequentes foram esvaziamento incompleto da bexiga (item 1), intermitência urinária (item 3) e esforço urinário (item 6), com frequência de respostas que indicavam ausência de sintomas de 51,5%, 54,5% e 63,3%, respectivamente. Além disso, a última questão do IPSS avalia a qualidade de vida das pessoas, que entre todos os participantes do estudo variou de boa a moderada. Na Figura 1 estão ilustradas as frequências dos sintomas considerando o escore total e nas diferentes intensidades (leve, moderada e grave).

**Figura 1:** Frequência dos sintomas do trato urinário inferior avaliados pelo IPSS.

Legenda: Item 1. Esvaziamento incompleto da bexiga; Item 2. Frequência urinária; Item 3. Intermitência urinária; Item 4. Urgência urinária; Item 5. Jato urinário fraco; Item 6. Esforço urinário; Item 7. Noctúria.

A investigação dos fatores associados à intensidade dos sintomas por meio do modelo de regressão binomial negativa evidenciou a influência de diferentes variáveis na piora da intensidade dos sintomas (Tabela 2).

**Tabela 2:** Modelo de regressão binomial negativa com as estimativas de efeitos das variáveis independentes na intensidade dos STUI avaliados pelo IPSS – São Carlos, SP, Brasil, 2023–2025.

Variáveis	Categoria da variável	β (EP)	*p*-valor	IRR	IC
Idade	–	0,04 (0,01)	<0,001	1,04	1,02–1,06
Cor	Brancos Pretos Pardos Outros	– 0,41 (0,17) 0,63 (0,16) 0,08 (0,53)	– 0,02 <0,001 0,86	– 1,88 1,51 1,09	1,35–2,62 1,07–2,15 0,37–3,10
História de câncer na família	Sim Não	-0,21 (0,11) –	0,07 –	0,81 –	0,63–1,03 –
Escolaridade	Ensino fundamental Ensino médio Ensino superior	– 0,32 (0,12) 1,23 (0,30)	– 0,01 <0,001	– 1,39 3,43	– 1,05–1,84 1,95–6,11
Consumo de bebida alcoólica	Sim Não	-0,30 (0,13) –	0,02 –	0,77 –	0,56–0,96 –
Hipertensão arterial	Sim Não	0,25 (0,13) –	0,06 –	1,29 –	0,97–1,70 –
Peso	–	0,01 (0,00)	0,01	1,01	1,00–1,02
Filhos	Sim Não	-0,49 (0,32) –	0,13 –	0,61 –	0,33–1,13 –
Ocupação	Empregados Desempregados Aposentados	– -0,40 (0,26) -0,54 (0,21)	– 0,12 0,001	– 0,67 0,58	– 0,39–1,14 0,38–0,87
Atividade remunerada	Sim Não	0,32 (0,13) –	0,01 –	1,38 –	1,05–1,82 –

β = Valor de estimativa, (EP) = Erro padrão, IRR = Razão de taxa de incidência, IC = Intervalo de confiança. Critério stepwise para seleção de variáveis. Coeficiente de determinação total: -1,12. Intercepto (Erro padrão): -0,81 (0,80); p > 0,05.

Os resultados da análise de regressão evidenciaram que o aumento da idade esteve diretamente associado à intensificação dos sintomas urinários, sendo que cada ano adicional representou elevação média de 4% no escore do IPSS (IRR = 1,04; IC95%: 1,02–1,06; p < 0,001), os participantes que se declararam pardos e pretos apresentaram, respectivamente, escores 51% (IRR = 1,51; IC95%: 1,07–2,15; p < 0,001) e 88% (IRR = 1,88; IC95%: 1,35–2,62; p = 0,02) maiores em comparação aos brancos, as pessoas com maior escolaridade tiveram os sintomas de maior intensidade, tanto para as pessoas que apresentavam ensino médio (IRR = 1,39; IC95%: 1,05–1,84; p = 0,01) como ensino superior (IRR = 3,43; IC95%: 1,95–6,11; p < 0,001), em relação ao ensino fundamental. Além disso, o peso corporal (p = 0,01) e a realização de atividade remunerada (p = 0,01) também foram associados ao aumento da intensidade dos sintomas.

O consumo de bebidas alcoólicas apresentou redução de 27% no escore IPSS (IRR = 0,77; p = 0,02) e a ocupação laboral representou uma redução de 42,1% no escore IPSS em comparação aos participantes aposentados (p = 0,001).

Para identificação dos sintomas urinários moderados e graves o IPSS apresentou sensibilidade de 91,7% e especificidade de 68,4%. A força de acurácia para identificação da diferença na intensidade dos sintomas urinários foi confirmada com uma AUC de 0,865 (Figura 2).

**Figura 2:** Curva ROC dos escores do IPSS em relação à discriminação dos níveis leves e moderados/graves.

## DISCUSSÃO

O presente estudo identificou que pessoas com condições prostáticas apresentaram níveis moderados de sintomas urinários e que a frequência urinária, jato urinário fraco e noctúria foram os principais sintomas apresentados. Os fatores associados ao aumento da intensidade dos STUI nesta população foram aumento da idade, tabagismo, peso corporal, a identificação em relação à cor parda ou preta, maiores níveis de escolaridade e exercício de atividade remunerada.

A intensidade moderada dos STUI é frequente na população com alterações prostáticas, quando avaliados pelo IPSS^(12,18)^, isto reforça a prevalência dos sintomas moderados em homens a partir dos 40 anos^(19)^. Este achado é relevante para que sejam propostas intervenções voltadas para o controle dos STUI entre os homens de maneira precoce, pois tais sintomas aumentam no decorrer do tempo e são associados ao comprometimento das atividades de vida diária^(19)^, da qualidade de vida e dos sintomas de ansiedade e depressão^(20)^.

A idade é um fator determinante para o agravamento das alterações miccionais, no presente estudo o aumento de um ano na idade dos participantes representou cerca de 4% de aumento do IPSS, ou seja, na intensidade dos sintomas. Estes achados estão alinhados aos de estudos realizados em diferentes países e com pacientes em condições clínicas semelhantes^(1,3,18)^. Ao considerar a longevidade da população, o aumento da prevalência dos sintomas com a idade, a baixa procura pelo cuidado e adequada avaliação dos sintomas, especialmente entre as pessoas com condições prostáticas^(21)^, ações e investimentos que estimulem o autocuidado com a saúde entre os homens são importantes e necessários.

A maneira como os participantes declararam sua cor foi um fator associado aos maiores níveis dos sintomas urinários, este dado compõe achado importante para direcionar a avaliação dos sintomas urinários nesta população, uma vez que as investigações e comparações sobre os STUI e as condições prostáticas entre diferentes grupos étnicos são limitadas e os estudos existentes se concentram nas populações com homogeneidade racial^(22)^.

A disparidade racial em relação às taxas mais elevadas de condições prostáticas e intensidade dos STUI entre pessoas negras é registrada na literatura, portanto é necessário que os estudos empreguem recortes populacionais diversos, para que ocorra redução de vieses acerca da avaliação entre distintos grupos raciais^(22)^, bem como no desenvolvimento de medidas efetivas para mitigar a desigualdade do cuidado no contexto geniturinário.

No cenário da pesquisa científica em saúde do homem, os achados deste estudo se alinham a investigações que apontam a alta prevalência de STUI em populações idosas, mas também ressaltam que fatores modificáveis, como o hábito de fumar e aumento do peso corporal, podem agravar sintomas urinários e intensificar o desconforto miccional^(6,21,23)^. Estes resultados possibilitam a incorporação de intervenções que consistem no controle de ambas as situações, com vistas à obtenção de resultados como redução dos incômodos provocados pelos STUI e melhora da qualidade de vida, por meio de ações que promovam mudança nos hábitos de vida.

Ainda assim, é relevante observar que, diferentemente de algumas pesquisas que demonstram relação consistente da hipertensão arterial e do diabetes mellitus com maior gravidade dos sintomas^(23)^, neste estudo tais variáveis não apresentaram associação estatisticamente significativa, o que reforça a importância de considerar o contexto local, as características do serviço de atendimento e o perfil clínico da população estudada.

A relação entre o consumo de bebidas alcoólicas e os sintomas do trato urinário inferior permanece inconsistente na literatura. Embora os achados deste estudo sugiram um possível efeito atenuante do álcool sobre os STUI, essa associação deve ser interpretada com cautela, pois pode refletir viés de confusão relacionado à ausência de distinção entre padrões de consumo moderado e abusivo. Na mesma linha deste resultado, evidências indicam que o consumo moderado de álcool pode estar associado à atenuação dos sintomas urinários^(24)^. Contudo, a mesma revisão sistemática demonstrou que o consumo abusivo de álcool está associado ao agravamento significativo dos STUI, comprometendo a função urinária, o que difere dos resultados observados nesta investigação^(24)^.

No presente estudo, além da idade e hábitos de vida, aspectos relacionados aos níveis educacionais e à situação laboral também foram fatores associados ao aumento da intensidade dos STUI. Estas condições se alinham à hipótese de que há uma relação da estabilidade psicológica, promovida pelos maiores níveis de escolaridade e renda, com a piora dos escores de STUI^(25)^. Estes achados demonstram a importância da avaliação da pessoa com STUI de maneira integral, considerando a totalidade dos determinantes sociais de saúde, pois o cuidado dos sintomas de intensidade moderada a grave volta-se às modificações do estilo de vida ao longo da vida e da manutenção de um status socioeconômico estável, e não por modificações da vida diária de curto prazo^(25)^.

Ainda, a relação encontrada nesta amostra, quanto aos maiores níveis educacionais e piora da intensidade dos sintomas, pode ser explicada pela probabilidade de existir uma melhor condição de compreensão do instrumento empregado na avaliação dos STUI, o que se caracteriza como viés de informação.

Tanto diretrizes clínicas como evidências científicas estimulam estratégias conservadoras no cuidado das pessoas com condições prostáticas^(1,26)^. Nesta abordagem, a Enfermagem tem papel fundamental, pois atua no gerenciamento desses sintomas, na identificação de fatores de risco, no aconselhamento individualizado e na promoção de mudanças comportamentais^(2,14)^. A atuação proativa do enfermeiro, baseada no Processo de Enfermagem, aliada ao acompanhamento contínuo e ao uso de estratégias educativas, potencializa a adesão do paciente às medidas propostas, favorecendo não apenas o alívio dos sintomas, mas também a melhoria da qualidade de vida e a prevenção de complicações associadas.

Deste modo, este estudo qualifica a atuação da Enfermagem como protagonista na investigação, avaliação e acompanhamento de homens com STUI, uma vez que esclarece o comportamento dos sintomas urinários e torna claros fatores demográficos e comportamentais que são responsáveis por influenciá-los. A literatura recente enfatiza que os enfermeiros que atuam na atenção primária podem desempenhar papel essencial no rastreamento, na orientação e na adesão de homens ao acompanhamento urológico^(26)^. Foi destacado na literatura que iniciativas de formação para o cuidado de Enfermagem em urologia contribuíram para consolidar protocolos de avaliação de continência e eliminação urinária no cenário brasileiro^(3)^.

Entre as principais contribuições científicas e práticas deste estudo está a oferta de evidências relevantes que fortalecem a etapa de Avaliação de Enfermagem^(27)^, por meio de melhor compreensão sobre as características dos sintomas urinários entre as pessoas com condições prostáticas, bem como dos seus fatores associados.

As limitações deste estudo centram-se no número amostral, no delineamento transversal e na seleção não probabilística dos participantes em um contexto hospitalar específico, fatores que podem introduzir viés de seleção, limitar a representatividade da amostra e restringir a generalização dos achados. A coleta de dados baseada em autorrelato também está sujeita a viés de informação, enquanto determinados resultados estatísticos devem ser interpretados com cautela em razão das limitações inerentes ao poder analítico do estudo. Além disso, a ausência de controle para potenciais variáveis confundidoras relevantes pode ter influenciado algumas associações observadas. Assim, mesmo o estudo reunindo achados importantes para o cuidado em urologia, as limitações supracitadas impossibilitam estabelecer relações causais entre as variáveis investigadas.

Para estudos futuros, recomenda-se o emprego de variáveis clínicas detectadas por exames complementares para triangulação metodológica e o acompanhamento dos pacientes por meio de desenhos de estudo longitudinal com intuito de acompanhar o efeito das variáveis investigadas na manifestação e intensidade dos sintomas urinários entre as pessoas com condições prostáticas.

Ademais, a ampliação de investigações que articulem o uso de instrumentos validados, a exemplo do IPSS, e tecnologias de apoio, como a ultrassonografia point-of-care (POCUS), que fortalecem a prática de Enfermagem nos serviços de saúde^(11,28,29)^ pode contribuir para o diagnóstico precoce, a estratificação de risco e a oferta de cuidados ainda mais resolutivos e personalizados para homens com condições prostáticas.

## CONCLUSÃO

O presente estudo alcançou seu objetivo ao identificar as especificidades dos sintomas do trato urinário inferior em pessoas com condições prostáticas atendidas em um serviço ambulatorial. Observou-se predominância de sintomas de intensidade moderada, com destaque para frequência urinária, jato fraco e noctúria.

A análise dos fatores associados evidenciou que idade, tabagismo, peso corporal elevado, maior escolaridade, exercício de atividade remunerada e cor da pele preta ou parda estiveram relacionados à maior gravidade dos sintomas, enquanto o consumo de bebidas alcoólicas e o vínculo formal de trabalho associaram-se à menor intensidade dos sintomas urinários.

Ademais, na população estudada, o IPSS demonstrou elevada sensibilidade e especificidade na distinção entre sintomas leves, moderados e graves, diferenciações clinicamente relevantes, apresentando excelente acurácia.

Estes resultados reforçam a importância de considerar fatores demográficos e comportamentais durante a avaliação de Enfermagem dos pacientes com condições prostáticas, pois apresentam relação com os sintomas urinários manifestados.

## Data Availability

Todo o conjunto de dados que dá suporte aos resultados deste estudo foi publicado no próprio artigo.

# Translation: Síntomas del tracto urinario inferior en personas con afecciones prostáticas: estudio exploratorio y transversal

**DOI:** 10.1590/1980-220X-REEUSP-2026-0116es

**Authors:** Pedro Henrique dos Santos Viana, Thiago Souza de Melo, Rafael Luis Bressani Lino, Aline Helena Appoloni Eduardo

**Affiliations:** 1 Universidade Federal de São Carlos, Programa de Pós-graduação em Enfermagem, São Carlos, SP, Brasil.; 2 Universidade Federal do Amazonas, Coari, AM, Brasil.; 3 Universidade Federal de São Paulo, Escola Paulista de Enfermagem, São Paulo, SP, Brasil.

**Keywords:** Síntomas del Sistema Urinario Inferior, Atención de Enfermería, Urología, Enfermedades de la Próstata

## Abstract

**Objetivo:**

Analizar las particularidades y los factores asociados a los síntomas del tracto urinario inferior en personas con afecciones prostáticas.

**Método:**

Estudio exploratorio y transversal realizado entre 2023 y 2025 en un hospital universitario del interior del estado de São Paulo. Participaron hombres mayores de 18 años con alteraciones prostáticas. Los datos se recopilaron mediante un formulario de caracterización y la Escala Internacional de Síntomas Prostáticos, y se analizaron mediante estadísticas descriptivas e inferenciales, con análisis exploratorios y regresión binomial negativa.

**Resultados:**

Se incluyeron 68 participantes, con una edad promedio de 66,7 años. Predominaron los síntomas de intensidad moderada (47,1 %); considerando el conjunto de los síntomas evaluados, el chorro débil, la frecuencia urinaria y la nicturia fueron los más prevalentes. La edad avanzada, un mayor nivel educativo, el peso corporal elevado y el color de piel negro o moreno se asociaron con una mayor gravedad, mientras que el consumo de alcohol y la ocupación laboral mostraron un efecto protector.

**Conclusión:**

Hubo predominio de los síntomas de almacenamiento y vaciamiento; se identificaron factores que influyen positiva y negativamente en la intensidad de los síntomas del tracto urinario inferior, lo que justifica la evaluación de enfermería.

## INTRODUCCIÓN

Los síntomas del tracto urinario inferior (STUI) representan manifestaciones clínicas frecuentes en los hombres, sobre todo entre aquellos con afecciones prostáticas, como la hiperplasia prostática benigna, la prostatitis crónica y el cáncer de próstata sospechoso o confirmado. Estos síntomas repercuten directamente en la calidad de vida, la autopercepción de la salud, la morbilidad psicológica y los costos en salud, tanto individuales como gubernamentales^(1,2)^.

Además, como consecuencia de los STUI, existe un mayor riesgo de caídas entre las personas de la tercera edad, un mayor riesgo de infección del tracto urinario debido al vaciamiento incompleto de la vejiga^(2)^, incomodidad emocional —especialmente como resultado de la incontinencia urinaria—, aislamiento social y descontento al lidiar con los síntomas^(3)^.

Desde el punto de vista clínico, los STUI tienen un origen multifactorial, son desencadenados por alteraciones orgánicas del tracto urinario inferior y se clasifican en síntomas de almacenamiento (urgencia, frecuencia, nicturia e incontinencia), de vaciamiento (hesitación, chorro débil, intermitencia y esfuerzo miccional) y posmiccionales (goteo terminal y sensación de vaciamiento incompleto)^(1)^.

Los principales factores para el desarrollo de los STUI en hombres incluyen el aumento del volumen prostático, la obstrucción uretral, alteraciones vesicales y factores no urológicos. Entre estos, se destacan las características no modificables, como el envejecimiento, y las modificables, como la obesidad, el síndrome metabólico y los hábitos de vida^(4)^.

Es importante conocer el impacto de estas condiciones modificables y del estilo de vida, ya que pueden contribuir al desarrollo de intervenciones de enfermería para controlar el origen no urológico de los STUI^(5)^, mediante actividades educativas que concienticen a las personas sobre la importancia de cambiar los hábitos que comprometen la eliminación urinaria, por ejemplo. Sin embargo, se observa una laguna en el conocimiento sobre los factores asociados capaces de agravar los STUI entre las personas con afecciones prostáticas.

Diferentes estudios poblacionales, realizados en Europa, Corea y Malasia, han evidenciado la alta prevalencia de los STUI en distintos países; los síntomas más comunes fueron la nicturia, el chorro débil y el goteo posmiccional(6–8). En Brasil, el estudio Brazil LUTS verificó, entre 2 751 hombres, que los síntomas más frecuentes fueron la nicturia, la frecuencia urinaria y el goteo terminal, además de destacar que la mayoría de los hombres presenta síntomas de intensidad moderada^(9)^.

Aunque no son condiciones exclusivas para el desarrollo de los STUI, las alteraciones prostáticas, especialmente la hiperplasia prostática benigna, se describen en la literatura como factores importantes asociados a la aparición de estos síntomas^(10)^. Sin embargo, la relación entre los STUI y el cáncer de próstata es controvertida, ya que algunos estudios señalan la existencia de una asociación entre estas variables, pero otros no han identificado una correlación significativa^(11,12)^.

La enfermería desempeña un papel central en la evaluación temprana y el manejo de los STUI, con intervenciones enfocadas en los ejercicios del suelo pélvico, el cateterismo y el asesoramiento sobre el estilo de vida^(1)^. En Irlanda, la implementación de clínicas dirigidas por enfermeros especialistas ha demostrado ser eficaz, segura y rentable, al ampliar el acceso ala atención urológica y evitar tratamientos inadecuados^(13,14)^. En Brasil, los estudios señalan intervenciones eficaces, pero también lagunas en el conocimiento y en la estandarización de la evaluación de los STUI por parte de los enfermeros^(15)^.

La atención de enfermería basada en el proceso de enfermería resulta rentable para las personas con STUI, ya que, a través de la evaluación de enfermería, se detectarán indicadores clínicos (factores de riesgo, signos y síntomas) que ayudan al enfermero a reconocer la alteración de la eliminación urinaria. Además, la evaluación de enfermería orientada a identificar indicadores clínicos específicos en personas con afecciones prostáticas permite la detección temprana de los síntomas iniciales, que suelen ser más sensibles a los tratamientos y cuidados aplicados, además de facilitar la derivación oportuna a otros profesionales de la salud, evitando retrasos en la atención. La demora en la identificación de casos más complejos o recurrentes puede provocar un agravamiento del cuadro clínico, pérdida de la función renal y una mayor demanda de exámenes diagnósticos e intervenciones de alto costo, lo que genera impactos significativos para el sistema de salud^(2)^.

En este contexto, la identificación de los síntomas más frecuentes en la población puede contribuir al manejo clínico y ayudar en la estratificación del riesgo de los pacientes con afecciones prostáticas.

Además, el análisis de la prevalencia de los síntomas y de los factores asociados a su intensidad puede favorecer una mayor precisión diagnóstica y mejorar la efectividad de las intervenciones terapéuticas.

De este modo, este estudio se basó en las siguientes preguntas de investigación: ¿Cuáles son los principales síntomas del tracto urinario inferior que presentan las personas con afecciones prostáticas y cuáles son los factores asociados al empeoramiento de la intensidad de los síntomas del tracto urinario inferior? Por lo tanto, el objetivo de este estudio fue analizar las características específicas y los factores asociados a los síntomas del tracto urinario inferior entre personas con afecciones prostáticas.

## MÉTODO

### Tipo de Estudio

Se trató de un estudio exploratorio y transversal, reportado de acuerdo con las directrices de Strengthening the Reporting of Observational Studies in Epidemiology (STROBE).

### Lugar del Estudio

El estudio se llevó a cabo en el Hospital Universitario adscrito a una institución pública de educación superior en un municipio del interior del estado de São Paulo. En el hospital, la investigación se realizó en el sector ambulatorio, que atiende diversas especialidades. Específicamente, el estudio se llevó a cabo en la clínica de urología general, que es un centro de referencia municipal en la atención y el seguimiento clínico en urología.

### Muestra y Criterios de Elegibilidad

Se incluyeron participantes mayores de 18 años, de sexo biológico masculino, que presentaban síntomas del tracto urinario inferior como consecuencia de alteraciones prostáticas, según el análisis realizado por los investigadores de los expedientes médicos para identificar el informe de STUI por parte del paciente o el registro médico. Se excluyeron los participantes que realizaban autocateterismo vesical o utilizaban una sonda vesical de permanencia. La selección de los participantes se realizó mediante muestreo de conveniencia, a partir de los pacientes previamente programados por la institución para atención ambulatoria.

### Procedimientos e Instrumentos para la Recolección de Datos

La recolección de datos se llevó a cabo entre octubre de 2023 y junio de 2025. Los pacientes fueron reclutados a medida que acudían a consultas ambulatorias, de manera electiva, con el urólogo. Tras la consulta médica, se invitaba a los participantes a formar parte del estudio y, ante su aceptación, el equipo de investigación realizaba la recolección de datos mediante entrevistas. Las entrevistas se llevaban a cabo en una sala reservada, ubicada en el consultorio ambulatorio, y tenían una duración de entre 20 y 30 minutos. Los instrumentos incluían información sociodemográfica y clínica, tal como se describe a continuación:

Formulario de caracterización sociodemográfica y clínica: elaborado por los autores, con el objetivo de identificar las variables sociodemográficas (edad, color de piel, estado civil, nivel educativo y situación laboral) y las variables clínicas (factores de riesgo para el desarrollo de enfermedades prostáticas, comorbilidades, cirugías e hospitalizaciones realizadas en el último año).

Escala Internacional de Síntomas Prostáticos (Inter­national Prostate Symptom Score – IPSS): evalúa la presencia de síntomas urinarios (frecuencia urinaria, urgencia urinaria, nicturia, intermitencia, chorro urinario débil, esfuerzo y vaciamiento incompleto), que suman un total de siete ítems. La frecuencia de aparición de cada síntoma se evalúa mediante una escala de Likert de seis puntos, en la que cero significa que el síntoma nunca se presenta y cinco que el síntoma se presenta casi siempre. El IPSS cuenta con una pregunta adicional sobre la calidad de vida del encuestado, que varía de uno a seis puntos, en la que la puntuación uno se asigna a la respuesta «excelente» y seis a «pésima». La puntuación final se obtuvo mediante la suma de los siete ítems sobre los síntomas urinarios y varía de cero a 35 puntos; la interpretación de los resultados se realizó mediante la siguiente estratificación: síntomas leves (de cero a siete puntos), moderados (de ocho a 19 puntos) y graves (de 20 a 35 puntos)^(16)^.

Este instrumento fue seleccionado por considerarse una herramienta esencial en la práctica clínica para la evaluación de los STUI y por ser ampliamente utilizado en estudios realizados en todo el mundo. Permite una cuantificación estandarizada de los síntomas del tracto urinario inferior, orienta el tratamiento, monitorea la progresión de la enfermedad y evalúa el impacto de los síntomas en la calidad de vida del paciente^(12)^. El IPSS fue validado para el contexto brasileño en una muestra de 281 personas con afecciones prostáticas, con una alta confiabilidad obtenida mediante el alfa de Cronbach, que fue de 0,816^(17)^.

### Aspectos Éticos

Para cumplir con las resoluciones del Consejo Nacional de Salud, el trabajo fue aprobado por el Comité de Ética en Investigación mediante el dictamen n.º 7.308.106 y el CAAE 82826624.2.0000.5504.

### Variables del Estudio y Análisis de los Datos

La variable dependiente del estudio fue la intensidad de los síntomas del tracto urinario inferior, medida mediante el IPSS, analizada tanto de forma categórica (síntomas leves, moderados y graves) como numérica (de 0 a 35 puntos), siendo las puntuaciones más altas indicativas de una mayor intensidad de los síntomas. Las variables independientes correspondieron a los factores sociodemográficos y clínicos obtenidos mediante el formulario de caracterización de los participantes.

Los datos se organizaron en una hoja de cálculo de Excel® y se analizaron con los programas SPSS® y R®. La normalidad de la variable dependiente se verificó mediante la prueba de Shapiro-Wilk (p > 0,05), adoptando análisis paramétricos. Las variables cuantitativas (continuas y discretas) se describieron mediante la media y la desviación estándar, mientras que las variables cualitativas (categóricas y nominales) se presentaron en frecuencias absolutas y relativas. La evaluación de la frecuencia de los síntomas del tracto urinario inferior se realizó mediante el análisis de los valores absolutos y los porcentajes, considerando las afecciones prostáticas presentes en la muestra y la intensidad de los síntomas urinarios identificada por el IPSS.

Para investigar las asociaciones entre las variables independientes y las puntuaciones del IPSS, se emplearon la prueba t de Student, el ANOVA y la correlación de Pearson. Al considerar el IPSS como variable categórica, se utilizaron las pruebas de Kruskal-Wallis, exacta de Fisher y Chi-cuadrado de Pearson. Además, se aplicó un modelo de regresión binomial negativa para estimar el efecto de las variables independientes sobre las puntuaciones del IPSS.

También se llevó a cabo un análisis de la curva ROC, mediante el área bajo la curva (area under the curve – AUC), para evaluar la precisión del IPSS en la identificación de síntomas leves y de síntomas moderados/graves, calculándose la sensibilidad y la especificidad. Se adoptó un nivel de significación del 5 % y un intervalo de confianza del 95 %.

## RESULTADOS

Participaron en el estudio 68 personas. Durante el proceso de reclutamiento, seis no fueron incluidas: una por negarse a participar (n = 1) y cinco porque no fue posible contactarlas en el momento de la espera para la consulta ambulatoria (n = 5).

La edad de los participantes varió entre 41 y 92 años, con un promedio de 66,7 años (desviación estándar ± 10,7). En la Tabla 1 se presentan todas las características de los participantes del estudio y las asociaciones con los resultados obtenidos en la evaluación de la intensidad de los síntomas del tracto urinario inferior.

**Tabla 1:** Caracterización de las variables sociodemográficas y clínicas de los participantes del estudio – São Carlos, SP, Brasil, 2023–2025.

Variables	IPSS total n = 68	*p* valor	IPSS			
			Leve n = 19	Moderado n = 32	Grave n = 17	*p* valor
	n (%)		n (%)	n (%)	n (%)	
**Edad (años)** Media (DE) < 60 años ≤ 60 años	66,7 (±10,7)	0,14^*^	04 (20,0) 15 (31,2)	14 (70,0) 18 (37,6)	02 (10,0) 15 (31,2)	0,02^**^
**Color de piel** Blancos Negros Mestizos Otro	42 (61,8) 10 (14,7) 15 (22,0) 01 (1,5)	**0,01** ^§^	14 (33,3) 03 (30,0) 01 (6,7) 01 (100)	19 (45,2) 05 (50,0) 08 (53,3) 00 (0,0)	09 (21,4) 02 (20,0) 06 (40,0) 00 (0,0)	0,31^§§^
**Estado civil** Casado Unión libre Divorciado Viudo Otro	50 (73,5) 02 (2,9) 06 (8,8) 01 (1,5) 09 (13,2)	0,10^§^	– 17 (34,0) 00 (0,0) 01 (16,7) 00 (0,0) 01 (11,1)	– 22 (44,0) 01 (50,0) 05 (83,3) 00 (0,0) 06 (47,1)	– 11 (22,0) 01 (50,0) 00 (0,0) 01 (100) 02 (25,0)	0,20^§§^
**Escolaridad** Primaria Enseñanza media Enseñanza superior	41 (60,3) 23 (33,8) 04 (5,9)	0,70^§^	13 (31,7) 06 (26,1) 00 (0,0)	18 (43,9) 11 (47,8) 03 (75,0)	10 (24,4) 06 (26,1) 01 (25,0)	0,77^§§^
**Ocupación** Empleado Desempleado Jubilado	17 (25,0) 04 (5,9) 47 (69,1)	0,93^§^	03 (17,6) 00 (0,0) 16 (34,0)	11 (64,7) 04 (100) 17 (36,2)	03 (17,6) 00 (0,0) 14 (29,8)	0,06^§§^
**Hijos** Si No	>65 (95,6) 03 (4,4)	0,88^++^	19 (29,2) 00 (0,0)	30 (46,2) 02 (66,7)	16 (24,6) 01 (33,3)	0,60^§§^
**Historia de cáncer de próstata en la familia** Si No	14 (20,6) 54 (79,4)	0,69^++^	02 (14,3) 17 (31,5)	09 (64,3) 23 (42,6)	03 (21,4) 14 (25,9)	0,51^$^
**Tabaquismo** Si No	29 (42,6) 39 (57,4)	**0,01++**	05 (17,2) 14 (35,9)	13 (44,8) 19 (48,7)	11 (37,9) 06 (15,4)	**0,05$**
**Consumo de bebida alcohólica** Si No	27 (39,7) 41 (60,3)	0,92^++^	05 (18,5) 14 (34,1)	16 (59,3) 16 (39,0)	06 (22,2) 11 (26,8)	0,18^$^
**Internación en último año** Si No	– 17 (25,0) 51 (75,0)	0,26^++^	– 04 (23,5) 15 (29,4)	– 07 (41,2) 25 (49,0)	– 06 (35,3) 11 (21,6)	0,62^§§^
**Cirugías anteriores** Si No	46 (67,6) 22 (32,4)	0,39^++^	13 (28,3) 06 (27,3)	19 (41,3) 13 (59,1)	14 (30,4) 03 (13,6)	0,22^§§^
**Actividad física** Si No	24 (35,3) 44 (64,7)	0,65^++^	07 (29,2) 12 (27,3)	12 (50,0) 20 (45,5)	05 (20,8) 12 (27,3)	0,80^$^
**Comorbilidad** Si No	48 (70,6) 20 (29,4)	0,58^++^	15 (31,2) 04 (20,0)	20 (41,7) 12 (60,0)	13 (27,1) 04 (20,0)	0,35^§§^
**Hipertensión arterial** Si No	33 (48,5) 35 (51,5)	0,43^++^	10 (30,3) 09 (25,7)	14 (42,4) 18 (51,4)	09 (27,3) 08 (22,9)	0,82^$^
**Cardiopatías** Si No	09 (13,2) 59 (86,8)	0,91^++^	01 (11,1) 18 (30,5)	06 (66,7) 26 (44,1)	02 (22,2) 15 (25,4)	0,77^$^
**Diabetes** Si No	25 (36,8) 43 (63,2)	0,72^++^	08 (32,0) 11 (25,6)	11 (44,0) 21 (48,8)	06 (24,0) 11 (25,6)	0,38^§§^
**Obesidad** Si No	54 (79,4) 14 (20,6)	0,42^++^	15 (27,8) 04 (28,6)	26 (48,1) 06 (42,9)	13 (24,1) 04 (28,6)	0,92^§§^
**Percepción de su salud** Excelente Buena Moderada Mala Muy mala	10 (14,7) 30 (44,1) 20 (29,4) 05 (7,4) 03 (4,4)	0,13^§^	06 (60,0) 07 (23,3) 04 (20,0) 01 (20,0) 01 (33,3)	02 (20,0) 16 (53,3) 10 (50,0) 02 (40,0) 02 (66,7)	02 (20,0) 07 (23,3) 06 (30,0) 02 (40,0) 00 (0,0)	0,44^§§^

^*^Prueba de correlación de Pearson

^§^ANOVA

^++^Prueba t

^**^Prueba de Kruskal-Wallis

^§§^Prueba exacta de Fisher

^$^Prueba de Chi-cuadrado de Pearson.

La media del IPSS fue de 13,5 (desviación estándar ± 8,9) y, en cuanto a la intensidad de los síntomas, 19 (27,9 %) pacientes presentaron síntomas leves, 32 (47,1 %) síntomas moderados y 17 (25,0 %) síntomas graves. En la muestra, solo un (1,5 %) participante obtuvo una puntuación de cero, lo que indica ausencia de síntomas urinarios, y también solo un participante obtuvo la puntuación máxima (35 puntos), lo que se clasifica como síntomas de intensidad grave y con la presencia de todos los síntomas evaluados por el IPSS. Por el contrario, 66 (97,0 %) participantes presentaron al menos un síntoma urinario.

Al analizar el resultado total de la puntuación obtenida mediante el IPSS, se observó que los síntomas más frecuentes fueron la frecuencia urinaria (ítem 2), el chorro débil (ítem 5) y la nicturia (ítem 7), con porcentajes de respuestas que indicaron la presencia de estos síntomas con un valor igual o superior al 75,0 %, mientras que los síntomas menos frecuentes fueron el vaciamiento incompleto de la vejiga (ítem 1), la micción intermitente (ítem 3) y el esfuerzo al orinar (ítem 6), con una frecuencia de respuestas que indicaban la ausencia de síntomas del 51,5 %, 54,5 % y 63,3 %, respectivamente. Además, la última pregunta del IPSS evalúa la calidad de vida de las personas, la cual, entre todos los participantes del estudio, varió de buena a moderada. En la Figura 1 se muestran las frecuencias de los síntomas considerando el puntaje total y en las diferentes intensidades (leve, moderada y grave).

**Figura 1:** Frecuencia de los síntomas del tracto urinario inferior evaluados mediante el IPSS.

Leyenda: Ítem 1. Vaciado incompleto de la vejiga; Ítem 2. Frecuencia urinaria; Ítem 3. Intermitencia urinaria; Ítem 4. Urgencia urinaria; Ítem 5. Chorro urinario débil; Ítem 6. Esfuerzo urinario; Ítem 7. Nicturia.

El análisis de los factores asociados a la intensidad de los síntomas mediante el modelo de regresión binomial negativa puso de manifiesto la influencia de diferentes variables en el empeoramiento de la intensidad de los síntomas (Tabla 2).

**Tabla 2:** Modelo de regresión binomial negativa con las estimaciones de los efectos de las variables independientes en la intensidad de los STUI evaluados mediante el IPSS — São Carlos, SP, Brasil, 2023–2025.

Variables	Categoría de la variable	β (EP)	*p*-valor	IRR	IC
Edad	–	0,04 (0,01)	<0,001	1,04	1,02–1,06
Color	Blancos Negros Mestizos Otros	– 0,41 (0,17) 0,63 (0,16) 0,08 (0,53)	– 0,02 <0,001 0,86	– 1,88 1,51 1,09	1,35–2,62 1,07–2,15 0,37–3,10
Antecedentes familiares de cáncer	Si No	-0,21 (0,11) –	0,07 –	0,81 –	0,63–1,03 –
Nivel educativo	Primaria Secundaria Superior	– 0,32 (0,12) 1,23 (0,30)	– 0,01 <0,001	– 1,39 3,43	– 1,05–1,84 1,95–6,11
Consumo de alcohol	Si No	-0,30 (0,13) –	0,02 –	0,77 –	0,56–0,96 –
Hipertensión arterial	Si No	0,25 (0,13) –	0,06 –	1,29 –	0,97–1,70 –
Peso	–	0,01 (0,00)	0,01	1,01	1,00–1,02
Hijos	Si No	-0,49 (0,32) –	0,13 –	0,61 –	0,33–1,13 –
Ocupación	Empleados Desempleados Jubilados	– -0,40 (0,26) -0,54 (0,21)	– 0,12 0,001	– 0,67 0,58	– 0,39–1,14 0,38–0,87
Actividad remunerada	Si No	0,32 (0,13) –	0,01 –	1,38 –	1,05–1,82 –

β = Valor estimado, (EE) = Error estándar, IRR = Razón de tasa de incidencia, IC = Intervalo de confianza. Criterio stepwise para la selección de variables. Coeficiente de determinación total: -1,12. Intercepto (Error estándar): -0,81 (0,80); p > 0,05.

Los resultados del análisis de regresión mostraron que el aumento de la edad se asoció directamente con la intensificación de los síntomas urinarios, y que cada año adicional representó un aumento promedio del 4 % en la puntuación del IPSS (IRR = 1,04; IC 95 %: 1,02–1,06; p < 0,001); los participantes que se identificaron como de raza mestiza y negra presentaron, respectivamente, puntuaciones un 51 % (IRR = 1,51; IC 95 %: 1,07–2,15; p < 0,001) y un 88 % (IRR = 1,88; IC 95 %: 1,35–2,62; p = 0,02) más altas, respectivamente, en comparación con los blancos; las personas con mayor nivel educativo presentaron síntomas de mayor intensidad, tanto en el caso de quienes contaban con educación secundaria (IRR = 1,39; IC 95 %: 1,05–1,84; p = 0,01) como de quienes contaban con estudios superiores (IRR = 3,43; IC 95 %: 1,95–6,11; p < 0,001), en comparación con quienes tenían estudios primarios. Además, el peso corporal (p = 0,01) y el hecho de realizar una actividad remunerada (p = 0,01) también se asociaron con un aumento en la intensidad de los síntomas.

El consumo de bebidas alcohólicas se asoció con una reducción del 27 % en la puntuación del IPSS (IRR = 0,77; p = 0,02), y el hecho de estar trabajando representó una reducción del 42,1 % en la puntuación del IPSS en comparación con los participantes jubilados (p = 0,001).

Para la identificación de los síntomas urinarios moderados y graves, el IPSS mostró una sensibilidad del 91,7 % y una especificidad del 68,4 %. La precisión para identificar la diferencia en la intensidad de los síntomas urinarios se confirmó con un AUC (área bajo la curva ROC) de 0,865 (Figura 2).

**Figura 2:** Curva ROC de los puntajes del IPSS en relación con la discriminación entre los niveles leves y moderados/graves.

## DISCUSIÓN

El presente estudio identificó que las personas con afecciones prostáticas presentaron niveles moderados de síntomas urinarios y que la frecuencia urinaria, el chorro urinario débil y la nicturia fueron los principales síntomas observados. Los factores asociados al aumento de la intensidad de los STUI en esta población fueron el aumento de la edad, el tabaquismo, el peso corporal, la identificación con la raza parda o negra, mayores niveles de escolaridad y el ejercicio de una actividad remunerada.

La intensidad moderada de los STUI es frecuente en la población con alteraciones prostáticas, cuando se evalúan mediante el IPSS^(12,18)^, lo que refuerza la prevalencia de los síntomas moderados en hombres a partir de los 40 años^(19)^. Este hallazgo es relevante para proponer intervenciones orientadas al control precoz de los STUI entre los hombres, ya que dichos síntomas aumentan con el paso del tiempo y se asocian con el deterioro de las actividades de la vida diaria^(19)^, de la calidad de vida y de los síntomas de ansiedad y depresión^(20)^.

La edad es un factor determinante para el agravamiento de las alteraciones miccionales; en el presente estudio, el aumento de un año en la edad de los participantes representó un incremento de aproximadamente el 4 % en el IPSS, es decir, en la intensidad de los síntomas. Estos hallazgos concuerdan con los de estudios realizados en diferentes países y con pacientes en condiciones clínicas similares^(1,3,18)^. Al considerar la longevidad de la población, el aumento de la prevalencia de los síntomas con la edad, la escasa búsqueda de atención médica y la evaluación adecuada de los síntomas, especialmente entre las personas con afecciones prostáticas^(21)^, son importantes y necesarias las medidas e inversiones que estimulen el autocuidado de la salud entre los hombres.

La forma en que los participantes declararon su color de piel fue un factor asociado a mayores niveles de síntomas urinarios; este dato constituye un hallazgo importante para orientar la evaluación de los síntomas urinarios en esta población, ya que las investigaciones y comparaciones sobre los STUI y las afecciones prostáticas entre diferentes grupos étnicos son limitadas, y los estudios existentes se concentran en poblaciones con homogeneidad racial^(22)^.

La disparidad racial en relación con las tasas más elevadas de afecciones prostáticas y la intensidad de los STUI entre las personas negras está documentada en la literatura; por lo tanto, es necesario que los estudios utilicen muestras poblacionales diversas, a fin de reducir los sesgos en la evaluación entre distintos grupos raciales^(22)^, así como para desarrollar medidas efectivas que mitiguen la desigualdad en la atención en el contexto genitourinario.

En el ámbito de la investigación científica sobre la salud del hombre, los hallazgos de este estudio concuerdan con investigaciones que señalan la alta prevalencia de los STUI en poblaciones de edad avanzada, pero también resaltan que factores modificables, como el hábito de fumar y el aumento de peso corporal, pueden agravar los síntomas urinarios e intensificar las molestias al orinar^(6,21,23)^. Estos resultados permiten incorporar intervenciones que consisten en el control de ambas situaciones, con el objetivo de lograr resultados como la reducción de las molestias provocadas por los STUI y la mejora de la calidad de vida, mediante acciones que promuevan un cambio en los hábitos de vida.

No obstante, es importante señalar que, a diferencia de algunos estudios que demuestran una relación consistente entre la hipertensión arterial y la diabetes mellitus con una mayor gravedad de los síntomas^(23)^, en este estudio dichas variables no presentaron una asociación estadísticamente significativa, lo que refuerza la importancia de considerar el contexto local, las características del servicio de atención y el perfil clínico de la población estudiada.

La relación entre el consumo de bebidas alcohólicas y los síntomas del tracto urinario inferior sigue siendo inconsistente en la literatura. Aunque los hallazgos de este estudio sugieren un posible efecto atenuante del alcohol sobre los STUI, esta asociación debe interpretarse con cautela, ya que puede reflejar un sesgo de confusión relacionado con la falta de distinción entre patrones de consumo moderado y abusivo. En la misma línea de este resultado, la evidencia indica que el consumo moderado de alcohol puede estar asociado con la atenuación de los síntomas urinarios^(24)^. Sin embargo, la misma revisión sistemática demostró que el consumo abusivo de alcohol está asociado con un empeoramiento significativo de los STUI, lo que compromete la función urinaria, lo cual difiere de los resultados observados en esta investigación^(24)^.

En el presente estudio, además de la edad y los hábitos de vida, los aspectos relacionados con los niveles educativos y la situación laboral también fueron factores asociados al aumento de la intensidad de los STUI. Estas condiciones concuerdan con la hipótesis de que existe una relación entre la estabilidad psicológica —promovida por mayores niveles de escolaridad e ingresos— y el empeoramiento de las puntuaciones de los STUI^(25)^. Estos hallazgos demuestran la importancia de evaluar de manera integral a las personas con STUI, tomando en cuenta la totalidad de los determinantes sociales de la salud, ya que el manejo de los síntomas de intensidad moderada a grave se centra en los cambios en el estilo de vida a lo largo de la vida y en el mantenimiento de una situación socioeconómica estable, y no en cambios a corto plazo en la vida cotidiana^(25)^.

Además, la relación observada en esta muestra entre los niveles educativos más altos y el empeoramiento de la intensidad de los síntomas puede explicarse por la probabilidad de que exista una mejor comprensión del instrumento utilizado en la evaluación de los STUI, lo que se caracteriza como un sesgo de información.

Tanto las guías clínicas como la evidencia científica promueven estrategias conservadoras en la atención de personas con afecciones prostáticas^(1,26)^. En este enfoque, la enfermería desempeña un papel fundamental, ya que interviene en el manejo de estos síntomas, en la identificación de factores de riesgo, en el asesoramiento individualizado y en la promoción de cambios de comportamiento^(2,14)^. La actuación proactiva del enfermero, basada en el Proceso de Enfermería, junto con el seguimiento continuo y el uso de estrategias educativas, potencia la adherencia del paciente a las medidas propuestas, lo que favorece no solo el alivio de los síntomas, sino también la mejora de la calidad de vida y la prevención de complicaciones asociadas.

De este modo, este estudio destaca el papel protagónico de la enfermería en la investigación, evaluación y seguimiento de hombres con STUI, ya que aclara el comportamiento de los síntomas urinarios y pone de manifiesto los factores demográficos y conductuales que influyen en ellos. La literatura reciente destaca que los enfermeros que trabajan en la atención primaria pueden desempeñar un papel esencial en la detección, la orientación y la adherencia de los hombres al seguimiento urológico^(26)^. Se ha resaltado en la literatura que las iniciativas de capacitación para la atención de enfermería en urología han contribuido a consolidar protocolos de evaluación de la continencia y la eliminación urinaria en el contexto brasileño^(3)^.

Entre las principales contribuciones científicas y prácticas de este estudio se encuentra la aportación de evidencia relevante que fortalece la etapa de evaluación de enfermería^(27)^, a través de una mejor comprensión de las características de los síntomas urinarios en personas con afecciones prostáticas, así como de sus factores asociados.

Las limitaciones de este estudio se centran en el tamaño de la muestra, el diseño transversal y la selección no probabilística de los participantes en un contexto hospitalario específico, factores que pueden introducir un sesgo de selección, limitar la representatividad de la muestra y restringir la generalización de los hallazgos. La recolección de datos basada en el autoinforme también está sujeta a sesgo de información, mientras que ciertos resultados estadísticos deben interpretarse con cautela debido a las limitaciones inherentes al poder analítico del estudio. Además, la falta de control de posibles variables de confusión relevantes podría haber influido en algunas de las asociaciones observadas. Por lo tanto, aunque el estudio reúne hallazgos importantes para la atención urológica, las limitaciones mencionadas anteriormente impiden establecer relaciones causales entre las variables investigadas.

Para estudios futuros, se recomienda el uso de variables clínicas detectadas mediante exámenes complementarios para la triangulación metodológica y el seguimiento de los pacientes a través de diseños de estudio longitudinales con el fin de evaluar el efecto de las variables investigadas en la manifestación y la intensidad de los síntomas urinarios entre las personas con afecciones prostáticas.

Además, la ampliación de investigaciones que articulen el uso de instrumentos validadoss, como el IPSS, y tecnologías de apoyo, como la ecografía point-of-care (POCUS), que fortalecen la práctica de la enfermería en los servicios de salud^(11,28,29)^, puede contribuir al diagnóstico precoz, a la estratificación del riesgo y a la oferta de cuidados aún más eficaces y personalizados para los hombres con afecciones prostáticas.

## CONCLUSIÓN

El presente estudio logró su objetivo al identificar las características específicas de los síntomas del tracto urinario inferior en personas con afecciones prostáticas atendidas en un servicio ambulatorio. Se observó un predominio de síntomas de intensidad moderada, entre los que se destacaron la frecuencia urinaria, el chorro débil y la nicturia.

El análisis de los factores asociados puso de manifiesto que la edad, el tabaquismo, el sobrepeso, un mayor nivel educativo, el ejercicio de una actividad remunerada y el color de piel negro o moreno se relacionaron con una mayor gravedad de los síntomas, mientras que el consumo de bebidas alcohólicas y el empleo formal se asociaron con una menor intensidad de los síntomas urinarios.

Además, en la población estudiada, el IPSS demostró una alta sensibilidad y especificidad para distinguir entre síntomas leves, moderados y graves, diferenciaciones clínicamente relevantes, con una precisión excelente.

Estos resultados refuerzan la importancia de considerar los factores demográficos y conductuales durante la evaluación de enfermería de los pacientes con afecciones prostáticas, ya que están relacionados con los síntomas urinarios manifestados.

## Data Availability

Todo el conjunto de datos que respalda los resultados de este estudio se publicó en el propio artículo.
